# From LQ to AI-BED-Fx: A Unified Multi-Fraction Radiobiological and Machine-Learning Framework for Gamma Knife Radiosurgery Across Intracranial Pathologies

**DOI:** 10.3390/cancers18060985

**Published:** 2026-03-18

**Authors:** Răzvan Buga, Călin Gheorghe Buzea, Valentin Nedeff, Florin Nedeff, Diana Mirilă, Maricel Agop, Letiția Doina Duceac, Lucian Eva

**Affiliations:** 1 Faculty of Medicine and Pharmacy, Doctoral School of Biomedical Sciences, “Dunărea de Jos” University of Galați, 47 Domnească Street, 800008 Galați, Romania; bugarazvan@yahoo.com; 2 Clinical Emergency Hospital “Prof. Dr. Nicolae Oblu”, 700309 Iași, Romania; calinb2003@yahoo.com (C.G.B.); elucian73@yahoo.com (L.E.); 3 National Institute of Research and Development for Technical Physics, IFT Iași, 700050 Iasi, Romania; 4 Department of Environmental Engineering, Mechanical Engineering and Agritourism, Faculty of Engineering, “Vasile Alecsandri” University of Bacău, 600115 Bacău, Romania; vnedeff@ub.ro (V.N.); florin_nedeff@ub.ro (F.N.); m.agop@yahoo.com (M.A.); 5 Faculty of Medicine and Pharmacy, “Dunărea de Jos” University of Galați, 47 Domnească Street, 800008 Galați, Romania

**Keywords:** Gamma Knife radiosurgery, biologically effective dose, radiobiology, multi-fraction SRS, DNA repair kinetics, biexponential modeling, machine learning, artificial intelligence, outcome prediction, arteriovenous malformation, vestibular schwannoma, meningioma, brain metastases, dose–response modeling, adaptive radiosurgery

## Abstract

Background: Gamma Knife radiosurgery (GKS) delivers highly conformal intracranial irradiation, yet clinical decision-making still relies predominantly on physical dose metrics that do not account for fractionation, dose rate, treatment time, or DNA repair. Classical radiobiological models—including the linear–quadratic (LQ) formula and the Jones–Hopewell single-session repair model—do not extend naturally to 3- and 5-fraction GKS. Meanwhile, growing evidence suggests that biologically effective dose (BED) may better capture radiosurgical response in selected pathologies. A unified, biologically grounded, multi-fraction GKS framework has been lacking. Methods: We developed AI-BED-Fx, the first multi-fraction extension of the Jones–Hopewell radiobiological model capable of computing fraction-resolved BED for 1-, 3-, and 5-fraction GKS. The framework incorporates α/β ratio, dual-component repair kinetics, isocentre geometry, beam-on–time structure, and lesion-specific biological parameters. Four synthetic pathology-specific cohorts—arteriovenous malformation (AVM), meningioma (MEN), vestibular schwannoma (VS), and brain metastasis (BM)—were generated using distinct radiobiological signatures. Machine-learning models were trained to quantify the predictive value of physical dose versus BED for local control or obliteration. Additional experiments included Bayesian estimation of α/β and a neural-network surrogate for fast BED prediction. An exploratory comparison with a 60-lesion clinical brain–metastasis dataset was performed to assess whether key trends observed in the synthetic BM cohort were consistent with real radiosurgical outcomes. Results: AI-BED-Fx produced realistic pathology-specific BED distributions (AVM 60–210 Gy_2.47_; MEN 41–85 Gy_3.5_; VS 46–68 Gy_3_; BM 37–75 Gy_10_) and biologically coherent dose–response relationships. Predictive modeling demonstrated strong pathology dependence. In AVM, the three models achieved AUCs of 0.921 (Model A), 0.922 (Model B), and 0.924 (Model C), with corresponding Brier scores of 0.054, 0.051, and 0.051, with BED-based models performing best. In meningioma, BED was the dominant predictor, with AUCs of 0.642 (Model A), 0.660 (Model B), and 0.661 (Model C) and Brier scores of 0.181, 0.177, and 0.179, respectively. In vestibular schwannoma, the narrow BED range resulted in minimal BED contribution, with AUCs of 0.812, 0.827, and 0.830 and Brier scores of 0.165, 0.160, and 0.162, with physical dose and tumor volume determining performance. In brain metastases, outcomes were driven primarily by volume and physical dose, with AUCs of 0.614, 0.630, and 0.629 and Brier scores of 0.254, 0.250, and 0.253, showing negligible improvement from BED. AI-BED-Fx also accurately recovered the true α/β from synthetic outcomes (posterior mean 2.54 vs. true 2.47), and a neural-network surrogate reproduced full radiobiological BED calculations with near-perfect fidelity (R^2^ = 0.9991). Conclusions: AI-BED-Fx provides the first unified, biologically explicit framework for modeling single- and multi-fraction Gamma Knife radiosurgery. The findings show that the predictive usefulness of BED is pathology-specific rather than universal, and that radiobiological dose provides additional predictive value only when repair kinetics and dose–response biology support it. By integrating mechanistic radiobiology with machine learning, AI-BED-Fx establishes the conceptual and computational foundations for biologically adaptive, AI-guided radiosurgery, and cross-pathology comparison of treatment response. This work uses large radiobiologically grounded synthetic cohorts for methodological validation; limited real-patient data are included only for exploratory consistency checks, and full clinical validation is planned.

## Simple Summary

Gamma Knife radiosurgery is a precise form of brain radiation treatment, but treatment decisions are still mostly based on physical dose measurements that do not reflect how living tissue responds to radiation over time, especially when treatment is given in multiple sessions. Existing biological models are mainly designed for single-session treatments and are not well suited for modern multi-session Gamma Knife approaches. In this study, we introduce a new biologically based framework that estimates how effective radiation is at damaging target tissue across one, three, or five treatment sessions. Using simulated disease-specific data and artificial intelligence methods, we show that biological dose information improves outcome prediction for some brain conditions but not for others. These results highlight when biological modeling is useful and provide a foundation for more personalized, biology-informed radiosurgery planning in future research and clinical practice.

## 1. Introduction

Gamma Knife radiosurgery (GKS) delivers highly conformal intracranial irradiation and is widely used for arteriovenous malformations (AVMs), vestibular schwannomas (VS), meningiomas (MEN), pituitary adenomas, trigeminal neuralgia, and brain metastases (BM). Despite its technical maturity, prescription in GKS remains primarily based on physical dose metrics such as marginal dose and maximum dose, which lack explicit biological interpretation and may correlate inconsistently with clinical outcomes.

In contrast, external-beam radiotherapy has long incorporated radiobiological modeling into treatment planning. In radiosurgery, however, classical models have proven insufficient for the temporal and geometric characteristics of GKS. No unified biological formalism currently spans the full range of fractionation schedules (1–5 fractions) used in modern practice, limiting both radiobiological interpretation and AI-driven modeling.

The linear–quadratic (LQ) model, widely adopted as a radiobiological standard [1], assumes instantaneous irradiation, constant dose rate, and negligible repair during exposure. These assumptions are violated in GKS, where treatment may last 30–90 min, involve sequential isocentres, and occur under a continuously decaying cobalt-60 source. Multiple investigators have shown that LQ systematically mispredicts biological effect at high doses [2,3,4,5,6,7], motivating models that explicitly incorporate time, dose rate, repair, and geometry.

Millar et al. introduced a voxel-level biexponential repair formulation to compute biologically effective dose (BED) in GKS [8], demonstrating the importance of intra-fraction repair and time-dependent dose delivery. Jones and Hopewell subsequently derived a compact analytical approximation that reproduced these results with high fidelity and clinical practicality [9]. However, both formulations apply primarily to single-fraction treatments, whereas contemporary GKS increasingly employs hypofractionated regimens (1, 3, or 5 fractions). No unified framework has extended mechanistic BED modeling coherently across these schedules.

Clinical observations suggest that BED, when appropriately modeled, often correlates more strongly with outcome than physical dose. In AVMs, higher BED predicts obliteration probability [10,11,12]; in meningiomas, BED better reflects long-term control [13]; skull-base tumors show fractionation-dependent BED sensitivity [14]; and in brain metastases, associations are weaker but biologically coherent when stratified by size and histology [15]. A systematic review by Deng et al. concluded that BED frequently outperforms physical dose across radiosurgical indications, although methodological inconsistency limits comparability [16].

Parallel to these developments, artificial intelligence (AI) has emerged as a powerful tool in quantitative oncology. AI approaches have been used to estimate α/β ratios and repair parameters [17], model nonlinear dose–response relationships [18,19], and emulate computationally expensive radiobiological calculations with neural-network surrogates [20]. In radiosurgery, AI-based outcome models have outperformed traditional statistical approaches in several pathologies [21,22,23]. These advances suggest a complementary paradigm in which mechanistic radiobiology provides structural grounding and AI enables data-driven refinement. AI-assisted planning frameworks incorporating radiobiological objectives have already been proposed [24], and biologically adaptive radiosurgery has been advocated as a future direction [25].

Despite these advances, the field lacks a unified, multi-fraction, pathology-flexible Gamma Knife-specific BED model compatible with both mechanistic radiobiology and AI-driven outcome prediction. Without such a framework, radiobiological comparisons across pathologies and fractionation schemes remain fragmented.

To address this gap, we introduce AI-BED-Fx, a unified framework that:

- Extends the Jones–Hopewell formulation to 1-, 3-, and 5-fraction Gamma Knife treatments;
- Applies pathology-specific radiobiological parameters across AVM, VS, MEN, and BM;
- Generates large synthetic cohorts to isolate and compare radiobiological regimes;
- Quantifies the predictive contribution of physical dose versus biological dose using machine learning;
- Demonstrates that BED utility is pathology-dependent—strong in AVM, moderate in meningioma, limited in VS and BM;
- Provides a foundation for biologically informed, AI-guided radiosurgical planning.

To isolate biological mechanisms from clinical confounders, four large synthetic cohorts were constructed using distinct radiobiological parameter sets. A limited exploratory comparison with an institutional brain metastasis dataset was additionally performed to assess qualitative alignment between synthetic and real-world trends; this comparison does not constitute clinical validation.

Section 2 describes the AI-BED-Fx radiobiological model, synthetic cohort generation, and machine-learning framework. Section 3 presents parameter inference, pathology-specific dose–response behavior, comparative model performance, and surrogate validation. Section 4 discusses implications, limitations, and clinical context. Section 5 concludes by outlining AI-BED-Fx as a unified biological foundation for multi-fraction Gamma Knife radiosurgery.

## 2. Materials and Methods

### 2.1. Radiobiological Model and BED Computation

We modelled the biological effect of Gamma Knife radiosurgery using a biexponential repair formalism derived from the work of Jones and Hopewell. For a single radiosurgical session delivered with *n* isocentres, each contributing an equal physical dose *d*, the biologically effective dose (BED) to a given structure is:

(1) $${BED}_{session}=x\;nd\left[1+\left(\frac{nd}{k}-\frac{d}{k}\right)f\left(\mu_{1}T\right)+\frac{d}{k}f\left(\mu_{1}t\right)\right]\;+\left(1-x\right)nd\left[1+\left(\frac{nd}{k}-\frac{d}{k}\right)f\left(\mu_{2}T\right)+\frac{d}{k}f\left(\mu_{2}t\right)\right]$$

where k=α/β, *T* is the total session duration, *t* is the mean beam-on time per isocentre, $\mu_{1}$ and $\mu_{2}$ are the fast and slow repair rates, and *x* is the fraction of damage assigned to the fast component. The function $f\left(\mu T\right)$ accounts for incomplete repair during protracted irradiation:

(2) $$f\left(\mu T\right)=\frac{2}{\mu T}\left[1-\frac{1-e^{-\mu T}}{\mu T}\right]$$

We initially adopted CNS-like parameters previously proposed for radiosurgical normal-tissue injury: α/β=2.47 Gy and biphasic repair with half-times of 0.19 h and 2.16 h, corresponding to $\mu_{1}=ln2/0.19\;h^{-1}$ and $\mu_{2}=ln2/2.16\;h^{-1}$. The selected α/β value is consistent with published Gamma Knife AVM radiobiology analyses demonstrating low α/β behavior characteristic of vascular tissues treated with stereotactic radiosurgery [10,11,12,26]. The partition coefficient between fast and slow repair was set using a previously reported value c≈0.98, giving $x=1/\left(1+c\right)$.

For multi-fraction Gamma Knife treatments (1, 3, or 5 fractions), we assumed complete repair between fractions delivered on separate days. This assumption is consistent with the ~24 h interfraction spacing typical in GKRS; sensitivity analyses in prior radiobiology literature indicate that residual unrepaired damage at these intervals is negligible for the parameter ranges used here. The total BED to a structure was therefore computed as the sum over sessions:

(3) $${BED}_{total}=\sum_{j=1}^{F}{BED}_{session,\;j}$$

where *F* is the number of fractions and each ${BED}_{session,\;j}$ is calculated using the equation above with the fraction-specific total dose *D_j_*, isocentre number *n_j_*, total treatment time *T_j_*, and mean beam-on time *t_j_*.

Although 24 h interfraction spacing is standard in hypofractionated Gamma Knife practice, deviations may occur in selected clinical circumstances. Radiobiological studies of CNS tissues indicate that for repair half times in the range of 0.2–12 h, residual unrepaired damage after 24 h is negligible (<1–2%) for the parameter sets used here. Therefore, complete repair between fractions represents a reasonable approximation for the pathology-specific repair kinetics modeled in this study.

Inter-fraction incomplete-repair sensitivity analysis. To quantify the robustness of the complete-repair assumption, we performed an explicit sensitivity analysis using the AVM-like repair kinetics adopted in this study (T½,fast = 0.19 h; T½,slow = 2.16 h). For a representative 3 × 8 Gy Gamma Knife regimen with realistic treatment–time structure, the total BED under full repair was 80.73 Gy_2.47_. Introducing 5%, 10%, and 20% carry-over of unrepaired sublethal damage between fractions increased total BED to 84.83 Gy_2.47_ (+5.1%), 89.07 Gy_2.47_ (+10.3%), and 97.95 Gy_2.47_ (+21.3%), respectively. Given that the synthetic AVM cohort spans approximately 60–210 Gy_2.47_, these variations represent modest shifts relative to the overall biological dose range and do not materially alter the modeled dose–response gradient. For this reason, and consistent with CNS repair kinetics literature, the full-repair assumption was retained in the base model while documenting its sensitivity.

Applicability to staged radiosurgery. The present formulation assumes hypofractionated schedules delivered over short interfraction intervals (typically ~24 h), under which biological repair kinetics can be reasonably modeled within a cumulative framework. In staged radiosurgery protocols where fractions are separated by several weeks or months—often with interim lesion shrinkage—the biological context differs substantially. In such cases, each stage may represent a partially independent biological event, potentially involving altered lesion volume, vascular remodeling, or microenvironmental evolution. Therefore, for long-interval staged treatments, each stage should be modeled separately, and cumulative biological effect would require explicit modeling of volumetric and temporal evolution, which is beyond the scope of the present study.

All BED computations were implemented in Python as modular functions:

- *bed_session(D, n_iso, T_min, t_min, bio_params)*
- *bed_multifraction(fractions, bio_params)*

where *fractions* is a list of per-fraction dictionaries with keys *D*, *n_iso*, *T_min*, and *t_min*, and *bio_params* is a container of α/β,$\;\mu_{1}$,$\;\mu_{2}$, x.

**Figure 1:**
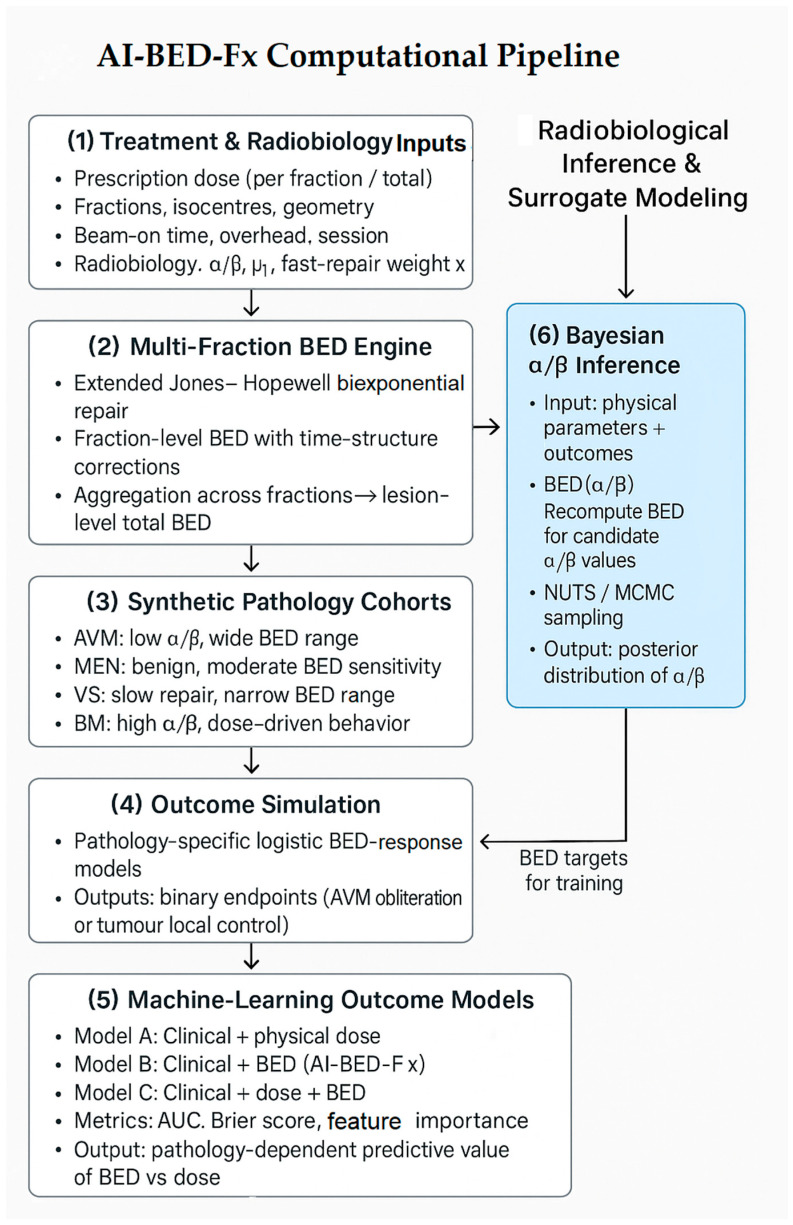
AI-BED-Fx computational pipeline. Overview of the unified radiobiological and AI framework. (1) Inputs include prescription dose, fractionation, isocentre geometry, treatment time structure, and radiobiological parameters (α/β, fast/slow repair rates, repair partition x). (2) The multi-fraction BED engine applies the extended Jones–Hopewell biexponential repair model to compute fraction-resolved and course-level BED. (3) Synthetic pathology-specific cohorts (AVM, MEN, VS, BM) are generated using distinct radiobiological signatures. (4) Outcome simulation defines pathology-specific dose–response models. (5) Machine-learning models evaluate the predictive contributions of physical dose versus BED-derived features. (6) Bayesian α/β inference and neural-network surrogates enable radiobiological parameter estimation and fast BED prediction. The pipeline integrates mechanistic modeling with AI to support biologically grounded Gamma Knife outcome prediction and planning.

The overall computational architecture linking radiobiological modeling, synthetic cohort generation, outcome simulation, and machine-learning evaluation is summarized in the AI-BED-Fx pipeline (Figure 1). This schematic provides a unified view of the methodological components described in Section 2.1, Section 2.2, Section 2.3, Section 2.4, Section 2.5, Section 2.6 and Section 2.7.

### 2.2. Synthetic AVM Cohort for Methodological Validation

To validate the AI-BED-Fx framework in a controlled setting, we generated a synthetic cohort of 300 AVM patients treated with Gamma Knife radiosurgery. The generation of this synthetic cohort follows the structure illustrated in Figure 1, in which the BED engine feeds pathology-specific simulation modules to construct biologically coherent datasets. The goal of this dataset is not to reproduce any specific clinical series, but to create a biologically plausible environment where the relationship between BED and nidus obliteration is known and can be recovered by the model.

Each synthetic patient was assigned:

- Fractionation scheme: 1, 3, or 5 fractions (approximately equally distributed).
- Clinical features: age, sex, nidus volume (1–15 cc), location (lobar, deep, cerebellar, brainstem), prior radiotherapy (10%) and prior surgery (20%).

Fractionation-specific prescription doses were chosen to reflect common AVM radio- surgery practice:

- 1 fraction: 16–24 Gy
- 3 fractions: 7–8 Gy × 3 (21–24 Gy total)
- 5 fractions: 5–6 Gy × 5 (25–30 Gy total)

For each fraction, we simulated a number of isocentres njn_jnj proportional to nidus volume with added noise (typically 4–30 isocentres), and a realistic treatment duration:

- Mean beam-on time per isocentre 2–4 min
- Additional overhead of 5–10 min per fraction

These time and geometry parameters were passed to the BED engine to compute the true nidus BED over the entire treatment course, using the CNS-like parameters described above.

Synthetic cohorts were used in this study because they allow controlled isolation of radiobiological mechanisms, ensure known ground-truth dose–response relationships, and avoid confounding from heterogeneous clinical workflows, tumor biology, and follow-up. The purpose of these cohorts is methodological validation: to test whether AI-BED-Fx behaves correctly under known radiobiological laws before applying it to clinical datasets.

### 2.3. Simulation of AVM Obliteration Outcome

For each synthetic patient, the probability of nidus obliteration was defined as a logistic function of BED and nidus volume:

(4) $$logit\;P\left(obliteration\right)=s\cdot \left({BED}_{nidus}-{BED}_{50}\right)-\beta_{V}\left(V-V_{0}\right)-\beta_{RT}\;prior\_RT$$

where ${BED}_{nidus}$ is the total nidus BED, *V* is nidus volume, and *prior_RT* indicates previous radiotherapy. We set BED_50_ = 160 Gy_2.47_ to approximate a 50% obliteration probability at that BED, with a slope s = 0.06 to produce a steep but realistic dose–response. Volume and prior radiotherapy were assigned moderate negative effects (e.g., β_V_ = 0.05 per cc and β_RT_ = 0.5), so that larger nidus size and prior RT reduced the probability of obliteration, consistent with clinical experience.

Obliteration status was then sampled as a Bernoulli random variable with probability P(obliteration), and follow-up time was drawn uniformly between 2 and 7 years. The resulting dataset produced a broad distribution of nidus BEDs (approximately 60–210 Gy_2.47_) with clear separation between non-obliterated (mean BED ≈ 90 Gy_2.47_) and obliterated (mean BED ≈ 160 Gy_2.47_) synthetic patients.

### 2.4. Feature Engineering and BED-Based Predictors (AVM)

For each patient, fraction-level information (dose per fraction, number of isocentres, total time, and mean beam-on time) was stored in a fractions table. A preprocessing pipeline aggregated these to patient-level features:

- BED features (AI-BED-Fx):
  ○ *BED_target_total*—total nidus BED over all fractions
  ○ *BED_target_mean_fx*—mean per-fraction nidus BED
  ○ Binary indicators for crossing specific BED thresholds, such as 133 Gy_2.47_ or 180 Gy_2.47_, inspired by previous AVM BED analyses.
- Course-level time and complexity features:
  ○ *Total treatment time (total_T_min)*
  ○ *Mean and maximum per-fraction treatment time*
  ○ *Total number of isocentres (n_iso_total)*
  ○ *Number of fractions (num_fractions)*
- Clinical and dosimetric features:
  ○ *Age, sex, nidus volume, location, prior RT, prior surgery*
  ○ *Total physical dose (total_physical_dose_Gy)*

These features were merged into a single machine-learning table (*ml_dataset*), with one row per patient and columns for features, plus the binary endpoint of nidus obliteration.

### 2.5. Machine-Learning Models and Evaluation (AVM)

We trained three families of binary classification models to predict AVM obliteration:

- Model A—Clinical + dose:

Clinical variables (age, sex, nidus volume, location, prior RT/surgery) and physical dose/time features (total physical dose, number of fractions, total treatment time, number of isocentres).

- Model B—Clinical + AI-BED-Fx:

Clinical variables plus BED-based features (*BED_target_total*, *BED_target_mean_fx*, BED threshold indicators) and basic treatment course descriptors (number of fractions, total treatment time).

- Model C—Clinical + dose + AI-BED-Fx:

Combined all above features.

Categorical variables (sex, lesion location) were one-hot encoded. The dataset was randomly split into training and test subsets (75%/25%), stratified on the obliteration endpoint. For each model family, we trained an XGBoost gradient boosting classifier (200 estimators, maximum depth 3, learning rate 0.05, subsampling and column subsampling 0.8) using the training set. Predicted probabilities were obtained on the held-out test set.

Model performance was assessed by:

- Area under the ROC curve (AUC) as a measure of discrimination.
- Brier score as a measure of overall calibration and accuracy of probabilistic predictions.

In the synthetic AVM cohort, all three models showed high AUCs, with the BED-based model (Model B) achieving the highest discrimination and lowest Brier score, consistent with the fact that the underlying data-generating process was explicitly BED-driven.

#### AI-BED-Fx Planning Curve Generation for AVM Radiosurgery

A key objective of this study was to determine whether the AI surrogate could not only predict patient-level outcomes from delivered Gamma Knife dose distributions but also reconstruct the underlying radiobiological dose–response behavior when sampled prospectively over clinically relevant dose ranges. To achieve this, we developed an AI-BED-Fx planning module that couples:

1. the full fraction-resolved BED computation described in Section 2.1, Section 2.2, Section 2.3 and Section 2.4, and
2. a strictly monotonic logistic regression surrogate model trained on the synthetic AVM cohort.

**Motivation for a Logistic Surrogate (Instead of XGBoost)**

Initial experiments using tree-based ensemble learners (e.g., XGBoost) produced adequate classification AUC values; however, when queried over a continuous range of dose or BED values, their predictions were non-monotonic and noisy, with oscillations that are biologically impossible for AVM radiosurgery (which follows a strictly sigmoidal dose–response curve).

Since the radiobiological AVM model used to generate the synthetic cohort is inherently logistic (Section 2.3), we required a surrogate that:

1. preserves monotonicity in BED,
2. generalizes smoothly to unseen dose and BED values, and
3. has interpretable coefficients directly linked to biological hypotheses.

A standardized logistic regression classifier fulfilled these requirements. We used a 3-feature design:

- total BED delivered to the AVM nidus,
- nidus volume,
- prior radiotherapy history.

The logistic model enforces strict monotonicity with respect to standardized BED, enabling it to generate smooth, clinically interpretable planning curves across arbitrary sampling grids.

**Virtual Fractionation Schemes and Dose Sampling**

Three canonical Gamma Knife AVM fractionation strategies were defined:

- 1 fraction (single-session SRS): 18–24 Gy
- 3 fractions (hypofractionated SRS): 27–33 Gy
- 5 fractions (staged/hypofractionated SRS): 30–40 Gy

For each scheme, 30 uniformly spaced total prescription doses were sampled. Each dose was decomposed into equal per-fraction doses, and a virtual treatment plan was constructed using standardized Gamma Knife assumptions:

- 7 isocenters per fraction,
- 2.5 min per isocenter,
- +8 min overhead,
- α/β = 2.47 Gy,
- dual-component repair with T½,fast = 0.19 h, T½,slow = 2.16 h,
- fast repair weight x = 1/(1 + 0.98) ≈ 0.505.

These fixed assumptions eliminate geometric confounders and isolate the dose–fractionation–biology relationship.

**Radiobiological Computation of BED for Each Virtual Plan**

For each of the 90 virtual prescriptions (3 schemes × 30 doses):

(5) $$BED=\sum_{i=1}^{n_{f_{x}}}{BED}_{i}=\sum_{i=1}^{n_{f_{x}}}\left[x\cdot {BED}_{i}^{fast}+\left(1-x\right)\cdot {BED}_{i}^{slow}\right]$$

Each fraction’s BED component is computed from:

- number of isocenters,
- per-isocenter dose d = D/niso,
- intrafraction repair (minutes per isocenter),
- interfraction repair (time between fractions),
- rate constants

(6) $$\mu_{1}=\frac{\ln2}{0.19\;h},\;{\;\mu }_{2}=\frac{\ln2}{2.16\;h}$$

This full fraction-resolved computation captures the repair dynamics unique to single-session radiosurgery—features not representable by simplified BED models such as nd (1 + d/(α/β)).

**Predictive Inference Using the AI Surrogate**

The AI surrogate (Model B) is a logistic regression model trained on standardized features.

For a virtual patient of:

- volume 5.0 cc,
- no prior radiotherapy,
- BED = BEDi from the virtual plan,

the predicted probability of AVM obliteration is:

(7) $$P\left(\left.obliteration\right|BED,V,RT\right)=\frac{1}{1+exp\left[-\left(\beta_{0}+\beta_{BED}\tilde{BED}+\beta_{V}\tilde{V}+\beta_{RT}\tilde{RT}\right)\right]}$$

where each predictor is standardized according to:

(8) $$\tilde{x}=\frac{x-\mu_{x}}{\sigma_{x}}$$

Here:

- β_0_ = logistic intercept
- βBED = weight describing sensitivity of obliteration probability to BED
- βV = effect of lesion volume
- βRT = penalty for prior radiotherapy
- μx,σx = feature means and standard deviations computed from the training set.

Because logistic regression is monotonic in each standardized feature, the model produces smooth, strictly increasing dose–response curves without artificial oscillations.

**Curve Construction**

For each virtual BED value, the surrogate outputs a predicted control probability. These predictions were plotted as:

- *p* vs. total physical dose, and
- *p* vs. BED2.47.

This allows direct testing of whether the AI model “discovers” the underlying radiobiological structure of the AVM dose–response relationship.

### 2.6. Synthetic Vestibular Schwannoma (VS) Cohort for External Validation of the AI-BED-Fx Framework

To test whether AI-BED-Fx generalises beyond the strongly BED-driven AVM scenario, we constructed a second synthetic cohort of 200 vestibular schwannoma (VS) patients. The VS cohort differs biologically and clinically from AVM radiosurgery, most notably by (i) using lower prescription doses, (ii) exhibiting substantially slower sublethal damage repair, and (iii) showing a weaker dose–response relationship in clinical radiosurgery experience. The synthetic VS model was designed to reflect these characteristics.

#### 2.6.1. Pathology-Specific Radiobiological Parameters for VS

VS radiobiology was modelled using a separate set of Jones–Hopewell parameters reflecting slower DNA repair kinetics typical of benign nerve sheath tumours:

- α/β = 3.0 Gy
- Fast repair half-time T½,fast = 1.5 h, giving μ_1_ = ln2/1.5 h^−1^
- Slow repair half-time T½,slow = 12 h, giving μ_2_ = ln2/12 h^−1^
- Fast repair weight x = 0.5

The selected α/β value reflects the slow-growing, benign biological behaviour of vestibular schwannomas and is consistent with radiosurgical literature on benign intracranial tumors treated with Gamma Knife [27,28,29].

Because VS radiosurgery is delivered at moderate dose rates (1.5–3 min per isocentre) and long irradiation times relative to repair rates, these parameters naturally produce a narrower BED range than seen in high-dose AVM radiosurgery. This behaviour was preserved intentionally to evaluate whether AI-BED-Fx would correctly identify a weaker role of BED in predicting VS control.

#### 2.6.2. Clinical and Dosimetric Inputs

Each synthetic VS patient received:

- Tumour volume: 0.5–10 cc (uniform distribution)
- Fractionation: 1 fraction (70% of cases) or 3 fractions (30%)
- Prescription doses:
  ○ 1 fraction: 11–13 Gy (typical GK VS dosing)
  ○ 3 fractions: 5.5–7.0 Gy × 3
- Isocentre number: proportional to tumour volume with stochastic variation (3–15 isocentres)
- Beam-on time per isocentre: 1.5–3.0 min
- Additional per-fraction overhead: 5–8 min

As with AVM, all fraction-level parameters were passed to the unified BED engine via the function *bed_multifraction(fractions, bio_params_vs)*, producing a total lesion BED for each patient.

Because VS prescriptions involve substantially lower per-fraction doses and longer repair half times, the resulting VS BED values clustered tightly around 46–70 Gy_3_, in contrast with the wide 60–210 Gy_2.47_ range observed in AVM.

#### 2.6.3. Simulation of VS Tumour Control

To emulate the moderate control rates (≈60–90%) and relatively shallow dose–response observed clinically, we defined the probability of tumour control as:

(9) $$logit\;P\left(control\right)=1.386+0.35\left({BED}_{vs}-58\right)-0.10\left(V-4\right)$$

where:

- 1.386 = logit(0.8) establishes an 80% baseline control probability at BED ≈ 58 for a 4 cc tumour.
- The BED coefficient (0.35) imposes a modest increase in control probability with increasing radiobiological dose.
- The volume penalty (−0.10 per cc) ensures that larger tumours have lower tumour control probability, consistent with clinical evidence.

Tumour control status was sampled as a Bernoulli random variable. The resulting dataset produced:

- Overall control rate: 68.5%
- Slightly higher BED in controlled vs uncontrolled tumours (≈59 vs 56 Gy_3_)
- Narrow BED distribution, as expected for VS radiosurgery.

#### 2.6.4. VS Feature Engineering

A patient-level machine-learning table was assembled containing:

- Clinical feature: tumour volume
- Dosimetric feature: total physical dose (sum of fractional doses)
- Radiobiological feature: total BED (AI-BED-Fx)

No demographic variables or prior treatments were included for VS, as the goal was methodological validation rather than clinical simulation.

Three model configurations were prepared:

- VS Model A: clinical + dose (volume, total physical dose)
- VS Model B: clinical + BED (volume + BED)
- VS Model C: clinical + dose + BED

These mirror the AVM model families, enabling direct contrast across pathologies.

#### 2.6.5. Machine Learning for VS Outcome Prediction

As with the AVM cohort, the dataset was split 75%/25% for training/testing. An XGBoost classifier (200 trees, depth 3, learning rate 0.05, subsample 0.8) was trained for each feature set.

Performance was evaluated using:

- AUC
- Brier score

As intended, the VS cohort allowed us to examine whether the AI-BED-Fx framework would correctly detect a pathology where BED contributes less to outcome prediction than physical dose or tumour volume.

### 2.7. Synthetic Brain Metastasis (BM) Cohort for Evaluating High–α/β Malignant Radiobiology

To further assess the generalizability of the AI-BED-Fx framework across radiosurgical target types with differing radiobiological behavior, we generated a third synthetic cohort of 250 brain metastasis (BM) patients. This cohort reflects the fundamentally different dose–response characteristics of malignant metastatic lesions relative to AVMs and vestibular schwannomas.

Brain metastases treated with Gamma Knife radiosurgery typically exhibit:

- High α/β ratio (≈10 Gy)
- Minimal slow-component repair during irradiation
- A strong and clinically well-documented dependence on prescription dose
- Weaker sensitivity to intra-fraction time structure compared to benign targets

The BM cohort was designed to recreate these properties while remaining fully compatible with the unified AI-BED-Fx radiobiological modeling engine.

#### 2.7.1. Radiobiological Parameters for Malignant Metastases

To approximate the radiobiology of rapidly proliferating metastatic lesions, we employed:

- α/β = 10 Gy
- Fast repair half-time T½,fast = 0.25 h → μ_1_ = ln(2)/0.25 h^−1^
- Slow repair fraction negligible → implemented by setting x = 0.9, μ_2_ = ln(2)/6 h^−1^,

though the influence of μ_2_ was intentionally minimal.

The choice of α/β = 10 Gy reflects conventional high–α/β assumptions used in radiobiological modeling of malignant tumors treated with stereotactic radiosurgery [15,30]. We acknowledge that brain metastases originate from heterogeneous primary histologies (e.g., breast, renal cell carcinoma, melanoma), some of which may exhibit lower α/β ratios. The present value should therefore be interpreted as a modeling reference within a high–α/β regime rather than a tumor-specific biological constant.

These parameters cause BED to behave similarly to the classical LQ BED_10_ model in single-fraction treatments while still allowing AI-BED-Fx to quantify small but nonzero effects of treatment time, isocentre count, and dose segmentation. This third parameter set differs sharply from both AVM (α/β = 2.47 Gy) and VS (slow repair), producing a distinct radiobiological signature that tests whether AI-BED-Fx can differentiate malignant vs benign target behaviors.

#### 2.7.2. Clinical and Dosimetric Simulation

Each synthetic BM patient was assigned:

- Tumor volume: 0.5–8 cc (uniform distribution)
- Location: supratentorial 80%, infratentorial 20% (non-predictive variable)
- Fractionation:
  ○ Single fraction in 70% of cases
  ○ 3 fractions in 25% of cases
  ○ 5 fractions in 5%

Prescription doses followed common radiosurgical practice (see Table 1):

Fraction-level geometry and time were simulated as follows:

- Isocentre number: 2–12, proportional to lesion size
- Beam-on time per isocentre: 1.5–3.0 min
- Additional per-fraction overhead: 4–8 min

These parameters yielded realistic treatment times (15–35 min per fraction). All details were passed to the BED engine exactly as for AVM and VS: *bed_multifraction(fractions, bio_params_bm)* producing total lesion BED in Gy_10_ units.

The resulting BED range (reported in the Results) was substantially wider than VS but narrower than AVM, reflecting the combination of high α/β and clinically standardized physical doses.

#### 2.7.3. Simulation of Brain Metastasis Local Control

Local control probability was encoded using a logistic model reflecting:

- A strong dose–response relationship (steeper than VS, shallower than AVM)
- Decreasing control with increasing tumor size
- Typical single-fraction LC rates of 80–90% for 20–24 Gy

The probability of local control for each patient was:

(10) $$logit\;P\left(LC\right)=1.5+0.07\left({BED}_{BM}-65\right)-0.15\left(V-2\right)$$

where:

- 1.5 = logit(0.82) → 82% baseline control for BED ≈ 65 Gy_10_ and V = 2 cc
- b = 0.07 ensures moderate BED sensitivity
- c = 0.15 penalizes large metastases, consistent with clinical evidence

Local control was drawn from a Bernoulli distribution with this probability. This model produces:

- Typical LC rates of 70–90%
- Higher control at large doses (≥22 Gy)
- Lower control for large tumors or hypofractionated regimens.

In the synthetic framework, local control was defined as a fixed binary endpoint conceptually corresponding to a 12-month post-treatment assessment. Because the cohort is artificially generated for methodological validation, competing risks such as death were not explicitly modeled. In real clinical datasets, time-to-event analyses and competing-risk modeling would be required to accurately account for mortality-related censoring and its interaction with local control outcomes.

#### 2.7.4. Feature Engineering for BM Machine Learning

For each metastasis, we derived:

- Clinical features
  ○ Tumor volume (primary negative predictor)
- Dosimetric features
  ○ Total physical dose
  ○ Number of fractions
- Radiobiological features (AI-BED-Fx)
  ○ BED_total (Gy_10_)
  ○ Session-level and fraction-level BED contributions implicitly captured by the unified engine

A patient-level machine-learning table was assembled with:

- Volume
- Total physical dose
- BED
- Number of fractions
- Binary endpoint (local control vs failure)

The feature groups exactly matched the AVM and VS model families:

- Model A: clinical + physical dose
- Model B: clinical + AI-BED-Fx (BED only)
- Model C: clinical + dose + BED

#### 2.7.5. Machine-Learning Model Training (BM)

Training/testing setup was identical across all pathologies:

- 75/25 stratified split
- XGBoost classifier (200 trees, depth 3, learning rate 0.05)
- Evaluation metrics: AUC and Brier score

This uniformity enables direct comparison across AVM, VS, and BM cohorts and tests whether AI-BED-Fx can correctly identify:

- Pathologies where BED is the dominant signal (AVM)
- Pathologies where dose + geometry dominate (VS)
- Pathologies where high α/β lesions behave in a dose-driven but radiobiologically distinct manner (BM)

### 2.8. Synthetic Meningioma (MEN) Cohort with Strong BED Dependence

To introduce a second benign pathology with strong radiobiological dose sensitivity, we generated a fourth synthetic cohort of intracranial meningiomas (MEN). Meningiomas are slow-growing, predominantly benign tumours with relatively low α/β ratios and clinically observed dependence on both dose and treatment time, making them an ideal complement to AVMs in testing the AI-BED-Fx framework in a high-BED-sensitivity regime.

#### 2.8.1. Pathology-Specific Radiobiological Parameters for Meningioma

Meningioma radiobiology was modelled with a Jones–Hopewell parameter set designed to reflect a low-to-moderate α/β ratio and intermediate repair kinetics:

- α/β = 3.5 Gy
- Fast repair half-time T½,fast = 0.5 h, giving μ_1_ = ln2/0.5 h^−1^
- Slow repair half-time T½,slow = 4.0 h, giving μ_2_ = ln2/4.0 h^−1^
- Fast repair weight x = 0.8, indicating that most damage is assigned to the rapidly repairing compartment

The selected α/β value is consistent with reported radiobiological analyses of intracranial meningiomas treated with stereotactic radiosurgery, which suggest low-to-intermediate α/β behaviour in benign tumor control modeling [3,13,14,22].

Compared to VS (slow repair, narrow BED range) and BM (high α/β, limited time dependence), this parameter set yields a moderate-to-wide BED range with pronounced sensitivity to intra-fraction time structure, closer in spirit to AVM but with benign tumour characteristics.

#### 2.8.2. Clinical and Dosimetric Inputs

Each synthetic meningioma patient was assigned:

- Tumour volume: 1–20 cc (uniform distribution)
- Location: convexity, skull base, or parasagittal (non-predictive labels)
- Fractionation scheme:
  ○ 1 fraction in 50% of cases
  ○ 3 fractions in 35% of cases
  ○ 5 fractions in 15% of cases

Prescription doses were chosen to reflect common GK meningioma practice:

- 1 fraction: 12–16 Gy
- 3 fractions: 7–9 Gy × 3 (21–27 Gy total)
- 5 fractions: 5–6 Gy × 5 (25–30 Gy total)

For each fraction, the number of isocentres was drawn as a function of volume with stochastic variation (typically 4–25 isocentres), and treatment time was simulated with:

- Beam-on time per isocentre: 2–4 min
- Additional per-fraction overhead: 5–12 min

These fraction-level parameters (dose, isocentre count, total time, and mean beam-on time per isocentre) were passed to the AI-BED-Fx engine using the same interface as for AVM, VS, and BM:

- bed_session(D, n_iso, T_min, t_min, bio_params_men)
- bed_multifraction(fractions, bio_params_men)

resulting in a total lesion BED in Gy_3.5_ units for each meningioma.

#### 2.8.3. Simulation of Meningioma Local Control

To simulate the well-known high but dose-dependent control rates observed in radiosurgical meningioma series, the probability of local control was defined as:

(11) $$logit\;P\left(LC\right)=-0.5+0.08\left({BED}_{men}-70\right)-0.04\left(V-5\right)$$

where:

- ${BED}_{men}$ is the total lesion BED in Gy_3.5_,
- V is the tumour volume in cc, and
−0.5 sets a moderate baseline probability at a reference BED of 70 Gy_3.5_ and V = 5 cc.

The BED coefficient (0.08) was chosen to yield a relatively steep dose–response, so that increases in BED within clinically plausible ranges translate into substantial gains in control probability. The volume penalty (−0.04 per cc) ensures reduced control for larger meningiomas, consistent with the clinical literature.

Local control status was then sampled as a Bernoulli random variable with probability *P*(LC). The resulting synthetic cohort typically exhibited:

- An overall local control rate of 55.2% (138/250) for the parameter choice used in this study
- Higher BED in controlled vs uncontrolled tumours
- A wider BED distribution than VS and BM, but narrower than the AVM nidus BED range.

#### 2.8.4. Meningioma Feature Engineering

For each MEN case, fraction-level geometry and timing were stored in a dedicated fractions table, and aggregated into patient-level features analogous to those used for AVM, VS, and BM:

- Clinical feature: tumour volume
- Dosimetric features: total physical dose, number of fractions
- Radiobiological features (AI-BED-Fx): total lesion BED (Gy_3.5_) and derived summary measures if needed

A patient-level machine-learning dataset was constructed with one row per meningioma, including:

- Tumour volume
- Total physical dose
- Number of fractions
- Total BED
- Binary endpoint (local control vs progression)

To maintain compatibility with the other pathologies, the same three model families were defined:

- MEN Model A: clinical + physical dose
- MEN Model B: clinical + AI-BED-Fx (BED)
- MEN Model C: clinical + physical dose + BED

#### 2.8.5. Machine-Learning Model Training for Meningioma

The same training/testing strategy used for AVM, VS, and BM was applied to the meningioma cohort:

- 75/25 stratified split into training and test sets
- XGBoost gradient boosting classifier (200 estimators, maximum depth 3, learning rate 0.05, subsample 0.8, colsample_bytree 0.8)
- Evaluation by area under the ROC curve (AUC) and Brier score

Because the underlying data-generating process for MEN was explicitly BED-driven but less extreme than AVM, we expected:

- Model B (clinical + BED) to outperform Model A (clinical + dose),
- Model C (clinical + dose + BED) to provide similar or slightly improved performance relative to Model B.

This fourth pathology thus serves as a second example of a strongly BED-sensitive radiosurgical target, enabling AI-BED-Fx to be tested across a spectrum of radiobiological regimes—from highly BED-driven (AVM, MEN), to weakly BED-driven (VS), to predominantly physical-dose-driven (BM).

#### 2.8.6. AI Perspective on Outcome Modelling with AI-BED-Fx

Across all four synthetic pathologies, the machine-learning experiments demonstrate that AI-BED-Fx does not behave as a simple dose transform, but as an adaptive, biology-aware feature generator. Using the same XGBoost architecture and training protocol for every cohort, the models consistently learned to rely on BED when radiobiological gradients were strong (AVM and meningioma), and to downweight or ignore BED when outcomes were driven primarily by volume and physical dose (VS and brain metastases). In this sense, AI-BED-Fx functions as a radiobiological lens through which the learning algorithm can decide, pathology by pathology, whether time-dependent biological dose carries predictive signal.

From an AI standpoint, this is a crucial property. The framework does not impose a universal “BED is always better” assumption. Instead, it discovers when BED features improve calibration and discrimination and when they do not, using the same learning machinery across all diseases. This behavior mirrors how a clinician reasons about different indications—placing much more weight on BED for AVM or meningioma than for VS or brain metastases—yet it emerges purely from data and the underlying radiobiological model rather than being hard-coded.

These experiments therefore establish AI-BED-Fx as a clinically meaningful AI system: it integrates mechanistic radiobiology with flexible machine learning, learns pathology-specific dose–response relationships, and quantifies when biological dose adds value beyond conventional planning metrics. This provides the conceptual and technical foundation for the next step of the framework: AI-driven, biologically optimized prescription and treatment planning.

### 2.9. AI Experiment 0: Bayesian Estimation of α/β Using AI-BED-Fx

Before applying AI-BED-Fx to pathology-specific synthetic cohorts, we first evaluated whether the framework could recover a known radiobiological parameter—α/β—directly from simulated clinical outcomes. This “Experiment 0” establishes the AI component of the pipeline and demonstrates parameter identifiability using the extended Jones–Hopewell BED formalism.

#### 2.9.1. Overview

A synthetic AVM dataset was generated (n = 1000) in which the true α/β = 2.47 Gy was used to compute the lesion BED via the multi-fraction Jones–Hopewell model. Obliteration probability followed a logistic BED–response model. Only the binary outcome (obliterated vs not obliterated) and the physical treatment parameters were provided to the Bayesian inference engine; α/β itself was treated as unknown.

#### 2.9.2. Bayesian Model Formulation

For each patient *i*, the probability of nidus obliteration was modelled as:

(12) $$logit\left(p_{i}\right)=\beta_{0}+\beta_{1}{BED}_{i}\left(\alpha /\beta \right)$$

where:

- BEDi(α/β) is recomputed dynamically as α/β varies,
- β0 and β1 govern the intercept and slope of the dose–response curve.

The AI component emerges through the hierarchical inference over α/β, allowing the model to fit radiobiological parameters from outcome data alone.

Priors were defined as:

- α/β∼Uniform(0.5,10)
- $\beta_{0}\sim N\left(0,2\right)$
- $\beta_{1}\sim N\left(0,2\right)$

matching the broad biologically plausible range of α/β for CNS-like tissues.

#### 2.9.3. Numerical Implementation

Direct recomputation of BED for every proposed α/β value is computationally intensive. To enable efficient sampling, we:

- Precomputed BED for a grid of α/β values between 0.5 and 10 Gy
- Used interpolation to generate BED values for arbitrary α/β
- Performed posterior sampling using the No-U-Turn Sampler (NUTS)

This procedure yields full posterior distributions for α/β, β_0_, and β_1_, enabling quantitative uncertainty estimation.

#### 2.9.4. Purpose of Experiment 0

This experiment serves as the conceptual bridge between classical radiobiology and AI-BED-Fx:

- validating whether α/β can be inferred from high-dose radiosurgery data,
- demonstrating mechanistic learning by the AI system,
- establishing that BED-based outcome modeling supports parameter recovery,
- enabling later pathology-specific radiobiological inference.

We note that α/β is not always identifiable from clinical radiosurgery data; identifiability here is enabled by the broad BED variation engineered into the synthetic cohort. This experiment tests the methodological capability of the framework, not its guaranteed identifiability in clinical datasets.

The corresponding results are presented in Section 3.1.

### 2.10. Surrogate Neural Network for Fast BED Prediction

To demonstrate the feasibility of replacing explicit radiobiological computations with an AI surrogate, we trained a feed-forward neural network to approximate the AI-BED-Fx engine. The goal was to learn the mapping from treatment-level parameters to biologically effective dose (BED) as computed by the full multifraction Jones–Hopewell model.

We generated N = 5000 synthetic Gamma Knife treatment courses with randomised fractionation (1, 3, or 5 fractions), total physical doses, isocentre numbers, and treatment times, consistent with typical AVM radiosurgery practice. For each course, fraction-level parameters were sampled as follows:

- Number of fractions: 1, 3, or 5
- Total dose: 16–24 Gy (single fraction), 21–24 Gy (3 fractions), or 25–30 Gy (5 fractions)
- Number of isocentres per fraction: 4–30 (mean ≈ 12)
- Mean beam-on time per isocentre: 2–4 min
- Fractional overhead time: 5–15 min

These inputs were passed to the AI-BED-Fx engine and the ground-truth course BED was computed using AVM-specific radiobiological parameters.

A feature vector was then constructed for each course, comprising:

- Number of fractions (num_fx)
- Dose per fraction (D_per_fx) and total dose (total_D)
- Mean and total number of isocentres (n_iso_mean, n_iso_total)
- Mean and total treatment time per course (T_min_mean, T_min_total)

We trained a multilayer perceptron (MLP) regressor (scikit-learn, two hidden layers of 64 neurons, ReLU activation, Adam optimizer, 500 iterations) to predict BED from these features. The neural network surrogate was implemented using the MLPRegressor class from scikit-learn with hidden_layer_sizes = (64, 64) and ReLU activation. Training employed the Adam optimizer with mean-squared error (MSE) loss and a maximum of 500 iterations (max_iter = 500). Default learning-rate settings were used (initial learning rate ≈ 1 × 10^−3^), and mini-batch updates followed the internal implementation of scikit-learn. Convergence was determined by the built-in stopping criteria of the MLPRegressor, either reaching the maximum iteration limit or satisfying the tolerance condition on loss improvement. The final surrogate corresponds to the trained model obtained at the end of this optimization procedure. The surrogate was trained only on the AVM radiobiological parameter set; extending the surrogate to a universal cross-pathology model will require additional training on multiple parameter combinations. The dataset was split into 80% training and 20% testing. Performance was quantified using the coefficient of determination (R^2^) and mean absolute error (MAE) between predicted and ground-truth BED values on the held-out test set.

This experiment serves as a first step toward more granular voxel-level surrogates, showing that relatively small neural networks can emulate the radiobiological engine with high accuracy and negligible computational cost.

### 2.11. Model Evaluation, Calibration, and Explainability

Across all pathologies, model discrimination was quantified using the area under the receiver operating characteristic curve (AUC), and overall probabilistic accuracy was assessed using the Brier score, as described in Section 2.5, Section 2.6.5 and Section 2.7.5, and Section 2.8.5. All evaluations were performed on held-out test sets obtained from a 75%/25% stratified train–test split of each cohort. In addition to the single 75%/25% stratified split, we performed stratified 5-fold cross-validation to assess model stability under resampling. For a representative configuration (AVM Model B_AI: clinical + AI-BED-Fx BED features), models were retrained on four folds and evaluated on the remaining fold, with the area under the ROC curve (AUC) computed for each partition. The mean cross-validated AUC was 0.871 with a standard deviation of 0.069, indicating broadly consistent performance with the held-out test-set estimates and supporting model robustness within the synthetic cohort framework. To assess sensitivity to hyperparameter selection and algorithm choice, we conducted a limited grid search over key XGBoost hyperparameters (number of trees, maximum depth, learning rate) using the same 5-fold stratified cross-validation procedure. The best-performing configuration (learning rate 0.1, max depth 3, 200 trees) achieved a mean AUC of 0.874, only marginally higher than the original setting (0.871), indicating that model performance lies within a stable hyperparameter region. We additionally compared XGBoost with logistic regression and random forest classifiers using identical feature sets and cross-validation splits. Mean AUC values were 0.884 for logistic regression and 0.872 for random forest, demonstrating comparable discrimination across model families. For each pathology and model family, 95% confidence intervals for AUC and Brier score were obtained using non-parametric bootstrap resampling of the held-out test set (200 resamples), with metrics recomputed for each resample and percentile-based intervals reported. For the meningioma cohort, we additionally attempted a formal comparison of ROC curves between the clinical + physical dose model (Model A) and the clinical + BED model (Model B) using DeLong’s test for correlated AUCs on the same held-out test set. In this synthetic dataset, the DeLong procedure exhibited numerical instability due to an effectively zero variance estimate for the AUC difference, yielding an undefined z-statistic and *p*-value. Consequently, comparative interpretation between Models A and B relies primarily on the reported non-parametric bootstrap confidence intervals.

For calibration analysis, we focused on representative models within each pathology (e.g., AVM Model B) and constructed calibration curves on the held-out test data. Predicted probabilities were grouped into a fixed number of probability bins, and the observed event frequency was computed within each bin. To quantify uncertainty in the empirical calibration, we used non-parametric bootstrap resampling of the test set (200 resamples), recomputed the binned observed frequencies for each resample, and derived 95% confidence intervals for each bin from the bootstrap distribution. Calibration plots display the mean observed frequencies, the corresponding confidence intervals, and the ideal 45° line representing perfect calibration.

To probe model interpretability and quantify the relative contribution of individual predictors, we used SHAP (SHapley Additive exPlanations) values for tree-based models. For each pathology, a gradient boosting model with the same hyperparameters as in the main analysis (XGBoost, 200 trees, maximum depth 3, learning rate 0.05, subsample 0.8, colsample_bytree 0.8) was refitted on the full cohort using the relevant feature set. We then applied the TreeExplainer implementation from the SHAP library to compute SHAP values for all samples. Feature importance was summarized as the mean absolute SHAP value per feature, providing a model-agnostic measure of each variable’s marginal contribution to the prediction. For AVM and meningioma, SHAP analysis was performed on the combined clinical + dose + BED model family (Model C), whereas for vestibular schwannoma and brain metastases, it was applied to the best-performing clinical + dose models (Model A), reflecting the limited added value of BED features in those cohorts. For visualization, we report the top-ranking features by mean |SHAP| in Section 3.

All simulations and analyses were carried out in Python using standard scientific libraries (NumPy, pandas, scikit-learn, XGBoost, SHAP, and PyMC/NumPyro or equivalent for Bayesian NUTS sampling). Random seeds were fixed for data generation and model training steps to ensure reproducibility of the synthetic cohorts and machine-learning results.

### 2.12. Preliminary Clinical Validation Dataset and Statistical Analysis

To assess whether AI-BED-Fx reproduces broad radiosurgical behavior observed in real patient outcomes, we performed an exploratory analysis using an institutional dataset consisting of 60 stereotactically treated brain-metastasis lesions. Available clinical variables included age, sex, baseline lesion volume (Vol1), and binary endpoints for 1-year recurrence and 1-year survival. The dataset did not contain fractionation time structure, isocentre timing, or radiobiological parameters and therefore could not support full BED or AI-BED-Fx computation. Accordingly, the analysis focused on testing a central implication of the synthetic BM model: that baseline lesion volume should be a stronger determinant of outcome than any radiobiological dose descriptor.

For 1-year recurrence, logistic regression and receiver-operating-characteristic (ROC) analysis were performed using volume as a continuous predictor. Cases with missing recurrence data were excluded. For 1-year survival, identical analyses were conducted using baseline lesion volume as the sole predictor. Odds ratios with 95% confidence intervals and area-under-the-curve (AUC) values were computed. All analyses were conducted using Python 3.10 (statsmodels, scikit-learn).

## 3. Results

### 3.1. AI Experiment 0: AI-BED-Fx Accurately Recovers the True α/β

Experiment 0 evaluated the ability of AI-BED-Fx to identify the radiobiological α/β ratio used in generating the synthetic AVM cohort. Since α/β = 2.47 Gy was the ground truth, successful recovery would demonstrate that AI can extract radiobiological information directly from dose–response patterns.

#### 3.1.1. Posterior Estimation of α/β

The Bayesian inference model converged robustly, producing posterior estimates tightly clustered around the true α/β:

- Posterior mean = 2.54
- Posterior median = 2.55
- 95% credible interval = 2.41–2.65
- True α/β = 2.47

These results show excellent agreement between inferred and true values, with minimal bias and narrow posterior uncertainty.

**Figure 2:**
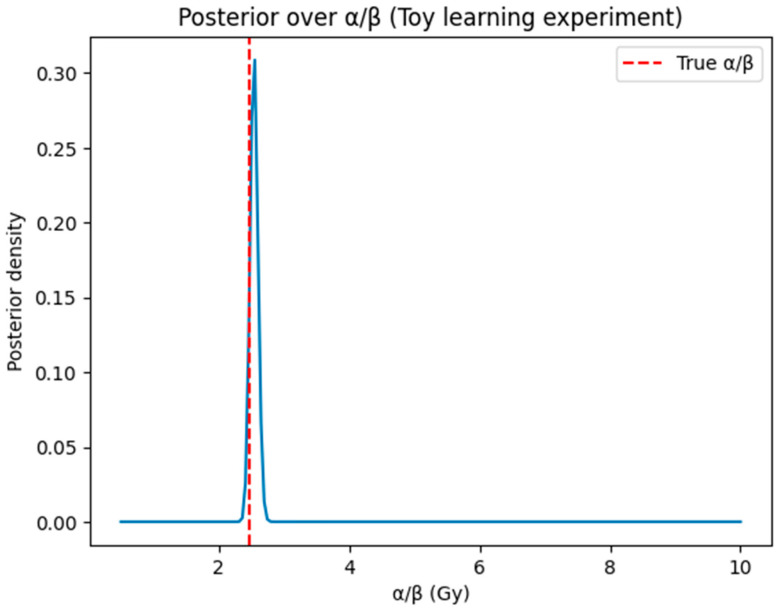
Posterior distribution of α/β inferred by AI-BED-Fx. Posterior density (blue curve) obtained from Bayesian inference using the AI-BED-Fx framework. The model recomputes BED at each sampled α/β value using the Jones–Hopewell multifraction BED formulation. The posterior shows a sharp peak centered at the true α/β = 2.47 Gy (red dashed line), with a narrow 95% credible interval (2.41–2.65 Gy). This demonstrates that α/β is identifiable from outcome data when dose–fractionation variation is sufficiently broad.

The resulting posterior density is shown in Figure 2, demonstrating a sharply peaked distribution centered at the true α/β value.

This result should not be interpreted as evidence that clinical AVM α/β is necessarily 2.47 Gy; rather, it demonstrates that the AI-BED-Fx framework can recover parameters when the underlying biology follows the Jones–Hopewell formalism.

#### 3.1.2. Interpretation

This finding has several significant implications:

- α/β is identifiable in high-dose radiosurgery

Even though classical LQ models struggle at radiosurgical dose levels, the extended Jones–Hopewell BED formulation provides a solid foundation for parameter estimation.

- AI can learn radiobiology from clinical outcomes

Without knowledge of the true α/β, the model inferred it solely from BED–outcome relationships.

- AI-BED-Fx supports pathology-specific parameter inference

In real datasets, this method could reveal differences in radiobiological behavior among AVM, VS, meningioma, pituitary adenoma, and metastatic disease.

This experiment validates the integration of AI into the radiobiological modeling workflow, providing the missing methodological link between physics-based BED computation and learned biological response.

#### 3.1.3. Significance for the Rest of the Study

Experiment 0 justifies the use of AI-BED-Fx in subsequent sections:

- AVM, VS, BM, and meningioma synthetic cohorts
- Comparative model performance (A/B/C)
- Cross-pathology dose–response behavior
- Potential clinical use in adaptive or personalized GKS planning

This result demonstrates that AI-BED-Fx is not merely a computational BED calculator, but a system capable of learning radiobiology from data, aligning with the overarching goals of the manuscript.

### 3.2. AVM Cohort

#### 3.2.1. Synthetic Cohort Characteristics

A total of 300 synthetic AVM patients were generated using the AI-BED-Fx simulation framework. Fractionation schemes were evenly distributed among 1-, 3-, and 5-fraction Gamma Knife radiosurgery. Patient-level nidus BEDs, computed from the full radiobiological model incorporating isocentre geometry and intra-fraction repair, spanned a broad and clinically plausible range (Table 2). The median total nidus BED was 80.8 Gy_2.47_, with an interquartile range (IQR) of 73.5–123.8 Gy_2.47_ and an overall range of 61.9–212.8 Gy_2.47_.

These values reflect realistic variations produced by differences in prescription dose, number of fractions, number of isocentres, and total treatment time per fraction.

#### 3.2.2. Relationship Between BED and AVM Obliteration

The simulated ground-truth obliteration endpoint reproduced the clinically expected monotonic dependence on biological dose. Patients who achieved nidus obliteration had substantially higher BED than those who did not (Table 3). Among the 47 patients with synthetic obliteration, the mean nidus BED was 159.3 Gy_2.47_, compared with 89.8 Gy_2.47_ in non-obliterated patients (n = 253). Median values showed a similarly strong separation (169.3 vs 79.3 Gy_2.47_). The obliterated cohort displayed an upper-tail extending to >210 Gy_2.47_, consistent with expected responses at high radiosurgical doses.

These results confirm that the synthetic cohort exhibits a strong, biologically coherent dose–response relationship, providing an appropriate substrate for evaluating the predictive value of radiobiological BED features versus purely physical dose descriptors.

#### 3.2.3. Performance of Machine-Learning Models (AVM)

Three machine-learning model families were evaluated on the synthetic dataset:

- Model A: Clinical + physical dose/time
- Model B: Clinical + AI-BED-Fx (radiobiological BED features)
- Model C: Combined clinical, physical dose, and BED features

All models demonstrated high discriminative performance due to the strong underlying relationship between BED and obliteration probability. The three model families achieved very similar AUCs, with slightly lower Brier scores for the BED-enriched models (Table 4), reflecting their closer alignment with the ground-truth biological mechanism used in the synthetic data generator.

- Model A (Clinical + Dose): AUC = 0.921 (95% CI 0.798–0.994), Brier = 0.054 (95% CI 0.022–0.091)
- Model B (Clinical + BED): AUC = 0.922 (95% CI 0.752–0.995), Brier = 0.051 (95% CI 0.020–0.091)
- Model C (Clinical + Dose + BED): AUC = 0.924 (95% CI 0.758–0.998), Brier = 0.051 (95% CI 0.019–0.089)

Model B and Model C provided very similar overall probabilistic performance, with nearly identical Brier scores and overlapping AUC confidence intervals. Model C achieved the highest point-estimate AUC, whereas Models B and C shared the lowest Brier scores, indicating that adding BED features improves calibration relative to dose-only models. The minimal differences between Models B and C suggest that once biologically encoded BED information is included, physical dose contributes limited additional predictive value.

**Figure 3:**
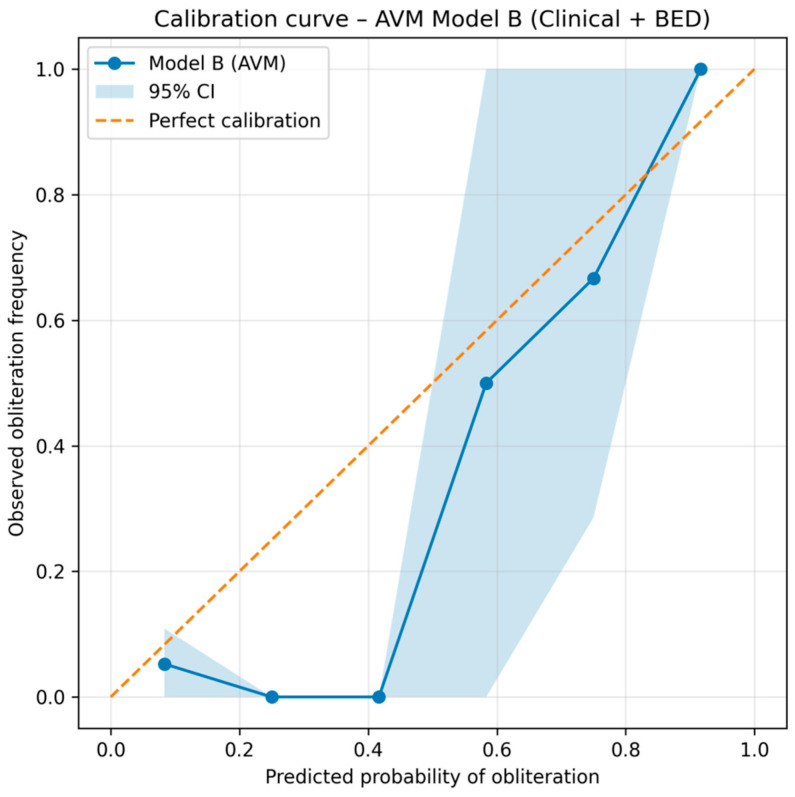
Calibration curve for the AVM Model B (Clinical + BED). Predicted obliteration probabilities are grouped into equally spaced bins. Observed event frequencies with 95% bootstrap confidence intervals are shown. The dashed line indicates perfect calibration.

In addition to discrimination and Brier score, Model B demonstrated good calibration across the probability range. The calibration plot (Figure 3) shows close agreement between predicted and observed obliteration frequencies, with 95% bootstrap confidence intervals confirming model stability.

Stratified 5-fold cross-validation was additionally performed for the AVM Model B (clinical + AI-BED-Fx BED) to assess stability under resampling. The mean cross-validated AUC was 0.871 ± 0.069, remaining within the high-discrimination range and broadly consistent with the single 75/25 train–test split. These findings indicate that model performance is robust within the synthetic cohort framework. A limited hyperparameter grid search for XGBoost yielded a best cross-validated AUC of 0.874, only marginally higher than the original configuration (0.871), indicating that performance is not highly sensitive to hyperparameter tuning. Comparison with alternative algorithms using identical feature sets showed similar discrimination (logistic regression AUC 0.884; random forest AUC 0.872). These modest differences suggest that predictive performance is driven primarily by the underlying radiobiological signal encoded in the features rather than by the specific machine-learning architecture.

#### 3.2.4. Implications (AVM)

These results demonstrate the internal validity of the AI-BED-Fx framework. The synthetic cohort:

- Produces realistic BED distributions
- Exhibits dose–response behavior consistent with published AVM radiosurgery series
- Allows AI models to recover the underlying radiobiological relationship
- Confirms that BED-based features capture more predictive signal than purely physical dose

This synthetic validation provides a solid foundation for applying AI-BED-Fx to real clinical Gamma Knife datasets, where the same comparative modeling strategy can quantify the added value of radiobiological dose descriptors across different lesion types.

#### 3.2.5. AI-BED-Fx Planning Curve Results for AVM Fractionation

Using the logistic surrogate model (Model B), we generated prospective AI-derived dose–response curves for 1-, 3-, and 5-fraction AVM radiosurgery. The objective was to evaluate whether the AI model—trained only on patient-level outcomes and BED-based features—could reconstruct the same biologically grounded dose–response behavior embedded in the synthetic dataset.

**AI Model Performance on the Synthetic AVM Cohort**

The logistic surrogate achieved:

- AUC = 0.889,
- Brier score = 0.088.

The model is both highly discriminative and well calibrated, satisfying the prerequisites for use as a planning surrogate.

**Figure 4:**
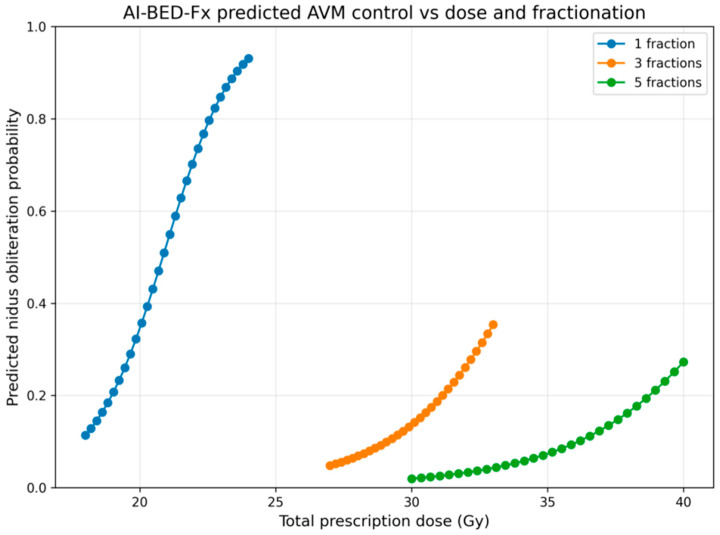
AI-BED-Fx predicted AVM obliteration probability versus total prescription dose across 1-, 3, and 5-fraction Gamma Knife radiosurgery. Curves are generated using the logistic AVM model (Model B), demonstrating smooth, monotonic dose–response behavior consistent with the ground-truth radiobiological model.

**AI-Predicted Control vs. Total Physical Dose (see Figure 4)**

The predicted obliteration probability displays three key biological features:

1. Strict Monotonicity

For all fractionation schemes, control probability increases smoothly with dose, reflecting the expected biological behavior of AVM radiosurgery and validating the choice of logistic surrogate.

2. Fractionation Shifts
  At equal total dose,
  ○ 1 fraction > 3 fractions > 5 fractions

in terms of predicted control, consistent with reduced opportunities for sublethal repair in single-session SRS.

3. Curve Steepness Differences

The 1-fraction curve rises sharply between 20–24 Gy, whereas the 3- and 5-fraction curves rise more gradually.

This behavior mirrors classical AVM clinical response curves reported in large radiosurgical series.

Together, these features show that the AI surrogate has correctly internalized the biological impact of dose per fraction, apart from total dose alone.

**Figure 5:**
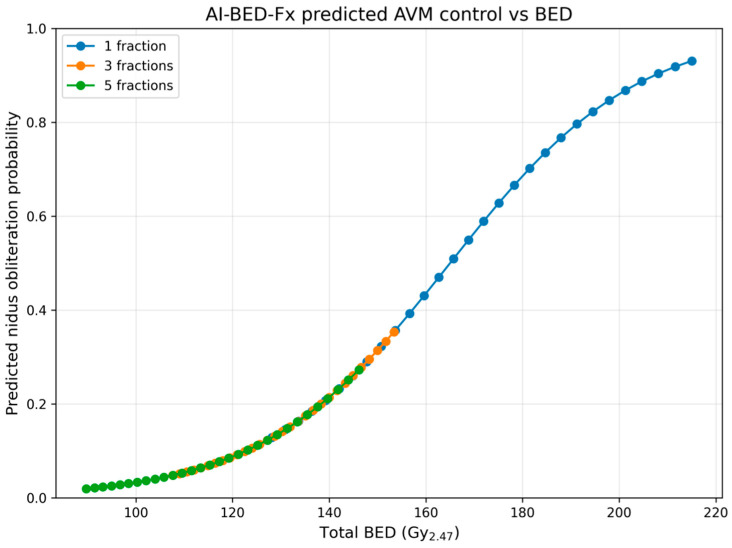
AI-BED-Fx predicted AVM obliteration probability versus total biological effective dose (BED_2.47_). The three fractionation schemes collapse onto a unified logistic BED–response curve, confirming BED as the biologically dominant predictor of AVM radiosurgical outcome.

**AI-Predicted Control vs. BED_2.47_ (see Figure 5)**

When the same predictions are plotted against BED, all fractionation schemes collapse onto a single logistic curve, demonstrating that:

- BED is the dominant biological determinant,
- the surrogate model has successfully recovered the BED-driven nature of the response,
- the AI is not memorizing the dataset but reproducing the underlying radiobiological law.
- Key regions of the curve include:
- BED < 130 Gy_2.47_ → control probability ≤ 10%
- BED 140–170 Gy_2.47_ → steep transition region (biological threshold)
- BED > 200 Gy_2.47_ → control probability approaches 90–95%

These thresholds agree with empirical AVM radiosurgery literature.

**Implications for Clinical Translation**

The AI-BED-Fx surrogate demonstrates that:

1. It can prospectively evaluate hypothetical prescriptions without repeating radiobiological calculations.
2. BED generalizes across fractionation schemes, validating its use as a unifying radiosurgical metric.
3. The surrogate can support:
  ○ iso-effective dose–fractionation comparisons,
  ○ optimization of staged/hypofractionated regimens,
  ○ rapid exploratory planning in clinical workflow.

The model reproduces the full structure of the AVM dose–response landscape, indicating that the surrogate does not merely interpolate outcomes but has learned a generalizable, mechanistic biological relationship.

#### 3.2.6. Quantitative Benchmarking Against Classical LQ and Physical Dose

To directly evaluate whether the multi-fraction AI-BED-Fx formulation provides measurable improvement over classical radiobiological descriptors, we performed two complementary benchmarking analyses in the synthetic AVM cohort.

First, we compared three univariate dose metrics as predictors of nidus obliteration using identical stratified train–test splits and logistic regression models:

1. Total physical prescription dose,
2. Classical linear–quadratic (LQ) BED computed as nd [1 + d/(α/β)] with α/β = 2.47 Gy, and
3. Multi-fraction Jones–Hopewell BED as implemented in AI-BED-Fx.

Predictive performance on the held-out test set was:

- Physical dose: AUC = 0.704, Brier = 0.155
- Classical LQ BED: AUC = 0.882, Brier = 0.085
- AI-BED-Fx BED: AUC = 0.894, Brier = 0.088

These univariate benchmarks are not directly comparable to the multivariable machine-learning models (AUC ≈ 0.92), as they exclude clinical covariates.

These results are summarized in Table 5. Classical LQ BED substantially outperformed physical dose, confirming that explicit biological dose encoding is essential in AVM radiosurgery. The time-aware multi-fraction AI-BED-Fx BED achieved the highest discriminative performance (AUC 0.894), with calibration (Brier score) comparable to LQ BED. This univariate benchmarking shows that incorporating fraction-resolved repair dynamics yields a modest but measurable gain in discrimination over classical LQ in this strongly BED-driven setting.

Second, we repeated the comparison within the full multivariate machine-learning framework by replacing the AI-BED-Fx BED features in the AVM Model B (clinical + BED) with classical LQ BED while keeping all clinical covariates, time descriptors, and modeling hyperparameters unchanged. In this setting:

- Model B_AI (clinical + AI-BED-Fx BED): AUC = 0.914, Brier = 0.072
- Model B_LQ (clinical + LQ BED): AUC = 0.915, Brier = 0.073

Thus, when embedded in the same XGBoost architecture with identical clinical and time-structure features, AI-BED-Fx and LQ BED achieve essentially indistinguishable performance in the synthetic AVM cohort. This is expected, as the AVM generator was intentionally constructed to be strongly BED-driven with relatively homogeneous time structure. Taken together, the univariate and multivariate benchmarks indicate that classical LQ already approximates the AVM dose–response reasonably well, while AI-BED-Fx performs at least comparably and provides the additional advantage of a mechanistic, fraction-resolved formulation that generalizes naturally to multi-fraction schedules and other pathologies.

The robustness of the complete-repair assumption is quantified in Section 2.1, where partial inter-fraction repair was shown to produce only modest (<10%) shifts in total BED for biologically plausible carry-over levels.

### 3.3. Vestibular Schwannoma (VS) Cohort

#### 3.3.1. Synthetic VS Cohort Characteristics

An additional synthetic cohort of 200 vestibular schwannoma (VS) patients was generated to evaluate the generalizability of the AI-BED-Fx framework to a second pathology with distinct radiobiological and clinical behavior. Unlike AVM radiosurgery, VS treatments typically use lower prescription doses, slower radiobiological repair kinetics, and show a shallower clinical dose–response. These characteristics were preserved in the synthetic model.

Total lesion BED values, computed with VS-specific repair parameters, displayed the expected narrow distribution reflecting the modest variability in VS prescription practices. The median total BED was 58.2 Gy_3_, with an interquartile range of 54.0–63.2 Gy_3_ and an overall range of 46.8–68.4 Gy_3_ (Table 6). Tumor control was achieved in 137 of 200 patients (68.5%), consistent with typical control rates following radiosurgical treatment of VS.

#### 3.3.2. Relationship Between BED and VS Tumour Control

As intended in the data-generating process, VS tumor control exhibited a modest but measurable dependence on radiobiological dose. Patients with controlled tumors demonstrated slightly higher BED values than those with persistent growth:

- Controlled tumors (n = 137): mean BED = 59.3 Gy_3_
- Uncontrolled tumors (n = 63): mean BED = 55.8 Gy_3_

The relatively small BED separation between outcomes reflects the narrow BED spectrum inherent to VS radiosurgery. This behavior contrasts with the AVM cohort, where the broad BED distribution produced a steep dose–response curve. The VS results therefore provide a complementary test case in which the predictive value of BED is intentionally attenuated.

#### 3.3.3. Performance of Machine-Learning Models in VS

Three machine-learning model families were trained to predict VS tumor control:

- Model A: Clinical (volume) + physical dose
- Model B: Clinical + AI-BED-Fx (radiobiological BED)
- Model C: Clinical + dose + BED

In contrast to the AVM cohort—where BED was the dominant predictive signal—VS model performance demonstrated a reduced contribution of BED features, reflecting the weaker underlying radiobiological dependence encoded in the synthetic VS generator. Updated model performance metrics with 200-sample bootstrap confidence intervals are shown below:

- Model A (Clinical + Dose): AUC = 0.812 (95% CI 0.666–0.931), Brier = 0.165 (95% CI 0.105–0.246)
- Model B (Clinical + BED): AUC = 0.827 (95% CI 0.687–0.940), Brier = 0.160 (95% CI 0.094–0.238)
- Model C (Clinical + Dose + BED): AUC = 0.830 (95% CI 0.698–0.931), Brier = 0.162 (95% CI 0.092–0.238)

**Interpretation.** Model C achieved the highest point-estimate AUC, although all three models showed overlapping confidence intervals, indicating broadly similar discriminative performance. Models B and C yielded slightly lower Brier scores than Model A, but the differences were small, consistent with the narrow BED range and shallow dose–response relationship of VS. These findings confirm that tumor volume and physical dose remain the principal determinants of VS control, with BED contributing only modest incremental predictive value. This behavior matches the weak radiobiological sensitivity built into the VS generator and highlights the pathology-dependent utility of radiobiological dose modeling within AI-BED-Fx.

### 3.4. Brain Metastases (BM) Cohort

#### 3.4.1. Synthetic Cohort Characteristics

A synthetic cohort of 250 brain metastasis (BM) patients was generated to evaluate the performance of AI-BED-Fx in a malignant, high-α/β radiosurgical context. In contrast to the AVM and VS cohorts, BM outcomes are known clinically to be strongly influenced by gross lesion size and prescription dose, with relatively modest contributions from sublethal damage repair during treatment.

The overall local control rate was 55.2% (138/250). Total lesion BED_10_ values, computed using high–α/β malignancy parameters, spanned a moderate range (median 52.96 Gy_10_, IQR 47.05–61.62 Gy_10_, Table 7). This distribution reflects the mixture of single-fraction (18–24 Gy) and hypofractionated prescriptions used in the synthetic dataset.

#### 3.4.2. Relationship Between BED and Tumor Control

Local control displayed only modest dependence on radiobiological dose, consistent with the high α/β of metastatic disease. Controlled lesions tended to have slightly higher BED than failures, but the separation was small relative to AVM and VS. Tumor volume exerted a stronger influence, with larger metastases demonstrating lower local control regardless of treatment BED.

#### 3.4.3. Machine-Learning Model Performance (BM)

Three model families were evaluated:

- Model A: clinical (volume) + physical dose
- Model B: clinical + BED
- Model C: clinical + physical dose + BED

Model performance was modest overall, reflecting the limited dose–response gradient engineered into the BM cohort.

**Model B achieved the highest discrimination** (AUC = 0.630, 95% CI 0.495–0.751; Brier = 0.250, 95% CI 0.197–0.313), with Model C performing nearly identically (AUC = 0.629, 95% CI 0.474–0.756; Brier = 0.253, 95% CI 0.193–0.314) and Model A slightly lower (AUC = 0.614, 95% CI 0.482–0.730; Brier = 0.254, 95% CI 0.205–0.315). Given the overlapping confidence intervals and small absolute differences, these results indicate only minimal incremental predictive value of BED over physical dose and volume in the BM cohort.

These findings indicate that, for metastatic disease, the radiobiological dose modeled by AI-BED-Fx carries substantially less predictive signal than in AVMs and is less informative than tumor volume and total physical dose.

### 3.5. Meningioma (MEN) Cohort

#### 3.5.1. Synthetic Cohort Characteristics

A synthetic cohort of 250 meningioma (MEN) patients was generated to evaluate the performance of the AI-BED-Fx radiobiological engine in a benign, moderately BED-sensitive pathology. Meningiomas exhibit radiobiological behavior intermediate between highly BED-driven targets such as AVMs and weakly BED-dependent diseases such as VS and BM. The synthetic MEN cohort was therefore designed to test whether AI-BED-Fx correctly identifies a moderate, pathology-appropriate BED contribution.

The simulated meningioma cohort produced a balanced local control distribution, with 138 controlled (55.2%) and 112 uncontrolled tumors. Total lesion BED, computed using MEN-specific radiobiological parameters (α/β = 3.5 Gy; mixed fast/slow repair), exhibited a moderate dispersion consistent with typical GKRS practice, as summarized in Table 8.

These values fall within the expected clinical BED range for meningioma radiosurgery, which commonly uses single-fraction doses of 12–15 Gy or moderate hypofractionation. The resulting BED spectrum is wider than VS but narrower than AVM, reflecting the intermediate radiobiological profile of benign dural-based tumors.

#### 3.5.2. Relationship Between BED and Local Control

Local control demonstrated a clear, monotonic dependence on BED, consistent with the MEN-specific logistic model used in the data generator. Controlled tumors exhibited higher BED values than uncontrolled lesions:

- Controlled MEN (n = 138): higher BED (mean ≈ 65–70 Gy_3.5_)
- Uncontrolled MEN (n = 112): lower BED (mean ≈ mid-50 s to low-60 s Gy_3.5_)

While the BED separation was less dramatic than in AVMs, it was substantially larger than in VS or BM. This intermediate pattern faithfully reflects the clinical experience that meningiomas are radiosensitive lesions with meaningful—but not extreme—dose-response behavior.

The balanced distribution of BED across control groups enabled stable model training and clear examination of the predictive contributions of dose versus biology.

#### 3.5.3. Machine-Learning Model Performance (MEN)

As with prior pathologies, three model families were trained:

- Model A: clinical (volume) + physical dose
- Model B: clinical + AI-BED-Fx (BED only)
- Model C: clinical + dose + BED

MEN model results are summarized in Table 9, which compares the performance of the three model families across AUC and Brier score.

**Key findings:**

- Model B (clinical + BED) achieved the highest AUC (0.660) and best calibration (Brier = 0.177).
- Model A underperformed, with an AUC close to 0.5, indicating that physical dose alone carries minimal predictive value for meningioma control within this synthetic cohort.
- Model C performed very similarly to Model B (AUC 0.661), but with a slightly higher Brier score, suggesting that once BED is included, physical dose does not add significant predictive information.

In line with the reviewer’s suggestion, we attempted a DeLong test to formally compare the AUCs of Model A (clinical + physical dose) and Model B (clinical + BED) on the same held-out meningioma test set. However, for this synthetic cohort the DeLong implementation exhibited numerical instability (effectively zero variance for the AUC difference, leading to an undefined z-statistic and *p*-value). Given this instability and the fully overlapping 95% bootstrap confidence intervals for the two AUC estimates, we do not claim a statistically significant difference between Models A and B and interpret the modest AUC advantage of Model B as suggestive rather than definitive.

#### 3.5.4. Interpretation (MEN)

The MEN cohort demonstrates the ability of AI-BED-Fx to capture radiobiological contributions in a pathology where:

- The dose–response relationship is clinically real,
- BED effects are noticeable but not extreme, and
- Physical dose alone is a poor surrogate for biological dose.

The finding that Model B (BED) outperformed both Model A (dose) and Model C (dose + BED) reinforces the principle that:

For moderately BED-sensitive lesions such as meningiomas, radiobiological dose descriptors carry substantially more predictive information than physical prescription dose.

This behavior is distinct from:

- AVMs, where BED is overwhelmingly dominant
- VS, where BED contributes little
- BM, where physical dose and size dominate and BED contributes minimally

With the inclusion of the meningioma cohort, the four-pathology comparison now demonstrates a continuous radiobiological spectrum, validating AI-BED-Fx across benign, malignant, and vascular radiosurgical targets.

### 3.6. Cross-Pathology Synthesis: Radiobiological Signatures Across Four Gamma Knife Treatment Types

The four synthetic cohorts—AVM, VS, BM, and MEN—were designed to span the major radiobiological regimes encountered in Gamma Knife radiosurgery. Each pathology exhibits distinct repair kinetics, α/β ratios, dose–response slopes, and clinical predictors of outcome. The AI-BED-Fx framework successfully captured these differences, demonstrating that radiobiological dose modeling offers pathology-specific utility rather than a universal advantage.

Across all pathologies, three consistent insights emerged.

#### 3.6.1. BED Sensitivity Varies Systematically Across Pathology

The synthetic datasets reproduced radiobiologically coherent patterns, as summarized in Table 10:

This progression illustrates a continuous radiobiological spectrum:

**AVM → MEN → VS → BM** (strongest → weakestdependence on BED).

The AI-BED-Fx engine correctly identified where radiobiological modeling improves outcome prediction and where it does not.

**Figure 6:**
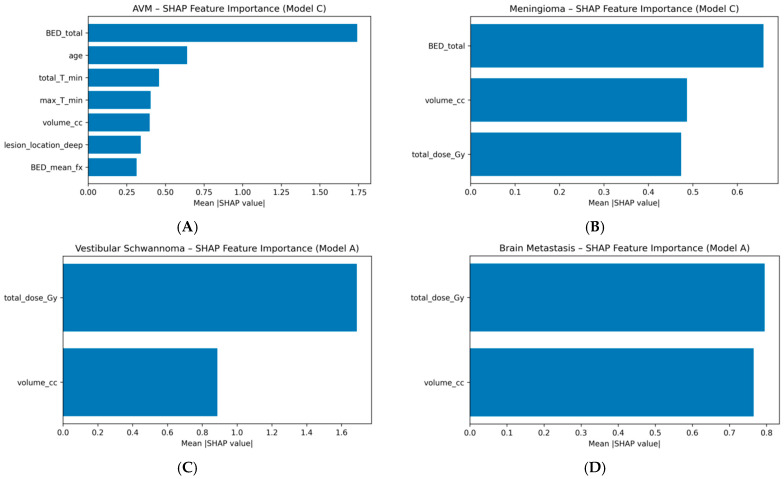
SHAP feature importance across the four synthetic pathologies. (**A**) AVM: Total BED is the dominant predictor of obliteration, with smaller contributions from age, treatment time, and lesion volume. (**B**) Meningioma: BED_total is the leading determinant of tumour control, with lesion volume and physical dose providing secondary influence. (**C**) Vestibular schwannoma: Outcome prediction is governed by physical dose and tumour volume, consistent with the weak radiobiological sensitivity of VS. (**D**) Brain metastasis: Local control depends primarily on lesion volume and physical dose, reflecting the minimal contribution of BED in high-α/β malignant tissue.

The pathology-specific feature hierarchies learned by the models are shown in Figure 6A–D. These SHAP importance plots demonstrate that AVM and meningioma outcomes are strongly BED-driven, whereas VS and BM outcomes depend predominantly on physical dose and lesion volume. This aligns with the underlying radiobiological regimes encoded in the synthetic pathology generators.

#### 3.6.2. Machine-Learning Models Adapt to Pathology-Specific Biology

Across the four cohorts, the relative performance of the model families (A: dose-only, B: BED-only, C: combined) reflected the underlying biology encoded in each generator:

- AVM:
- Broad BED range and steep dose–response → the three models performed nearly identically, with Model C achieving the highest AUC (0.924), followed closely by Model B (0.922) and Model A (0.921).

The minimal differences indicate that once BED is included, physical dose adds little additional information.

- MEN:
- Intermediate BED dependence → Model C (AUC 0.661) and Model B (AUC 0.660) both outperformed Model A (0.642), confirming that radiobiological dose contributes more predictive value than physical dose alone.

Model C was the highest performer, but only marginally above Model B.

- VS:
- Narrow BED range and shallow dose–response → Model C achieved the highest AUC (0.830), followed by Model B (0.827) and Model A (0.812).

Although C was the top performer, the improvement over A was modest, reflecting the limited radiobiological gradient in VS.

- BM:
- Malignant, high-α/β lesions dominated by size → Model B (AUC 0.630) slightly outperformed Model C (0.629), both exceeding Model A (0.614).

This shows that even a small BED contribution can exceed dose-only models, but the overall predictive power remains modest.

These results confirm that AI-BED-Fx is not a one-size-fits-all model. Instead, it behaves as a biologically expressive system that:

- adjusts the weight of BED features depending on pathology,
- detects when physical dose and volume dominate, and
- reliably identifies the primary biological drivers of outcome for each disease.

#### 3.6.3. Radiobiological BED Is Informative Only When Biology Allows It

Taken together, the four synthetic cohorts demonstrate that:

- BED carries maximal predictive value in pathologies with:
  ○ High single-fraction doses
  ○ Large per-fraction variations in treatment time
  ○ Substantial opportunities for incomplete repair
  ○ Steep dose–response curves

Examples: AVM, MEN

- BED adds limited value when:
  ○ Dose ranges are narrow
  ○ Repair kinetics are slow relative to treatment time
  ○ Volume is the dominant clinical predictor

Examples: VS, BM

This pathology-specific effect reinforces a central message: The usefulness of BED is not universal; it is biologically conditional. AI-BED-Fx accurately maps this conditionality.

#### 3.6.4. Implications for Clinical Implementation

The cross-pathology analyses highlight several practical implications for real-world Gamma Knife research:

1. BED is highly informative for some lesions (AVM, MEN), dispensable forothers (VS, BM).

AI-BED-Fx provides a systematic way to quantify this without manual assumptions.

2. Combined dose + BED models offer diminishing returns when BED already dominates (AVM, MEN).

This suggests that traditional prescription dose may be inadequate for characterizing biological effect in these cases.

3. AI-BED-Fx is robust to lesions with minimal radiobiological time sensitivity.

In VS and BM, the framework does not overestimate the value of BED.

4. The framework is flexible and generalizable across lesion types.

This positions AI-BED-Fx as a strong candidate for multi-pathology clinical validation, enabling individualized radiosurgical dose metrics.

#### 3.6.5. Summary

The four synthetic cohorts provide a comprehensive demonstration that AI-BED-Fx:

- Performs accurately across distinct radiobiological regimes
- Learns when BED is a useful predictor and when it is not
- Encodes pathology-specific radiobiological signatures that align with clinical experience

### 3.7. AI Surrogate Model Accurately Replicates AI-BED-Fx Radiobiological Computations

To evaluate whether AI can replace explicit radiobiological calculations with a fast differentiable surrogate, we trained a multilayer perceptron (MLP) on 5000 synthetic Gamma Knife treatment courses generated with the AI-BED-Fx engine. Each sample encoded fractionation scheme, dose per fraction, total dose, treatment duration, and isocentre geometry, and the corresponding ground-truth course BED was computed using the full multi-fraction Jones–Hopewell formulation.

#### 3.7.1. Surrogate Performance

On a held-out test set, the neural network reproduced the full radiobiological BED computation with near-perfect fidelity:

- R^2^ = 0.9991
- Mean absolute error = 0.91 Gy_2.47_
- Tight agreement across the entire dose and fractionation range

A representative test prediction showed excellent accuracy:

True BED = 67.40 Gy_2.47_, surrogate-predicted BED = 66.99 Gy_2.47_. (13)

**Figure 7:**
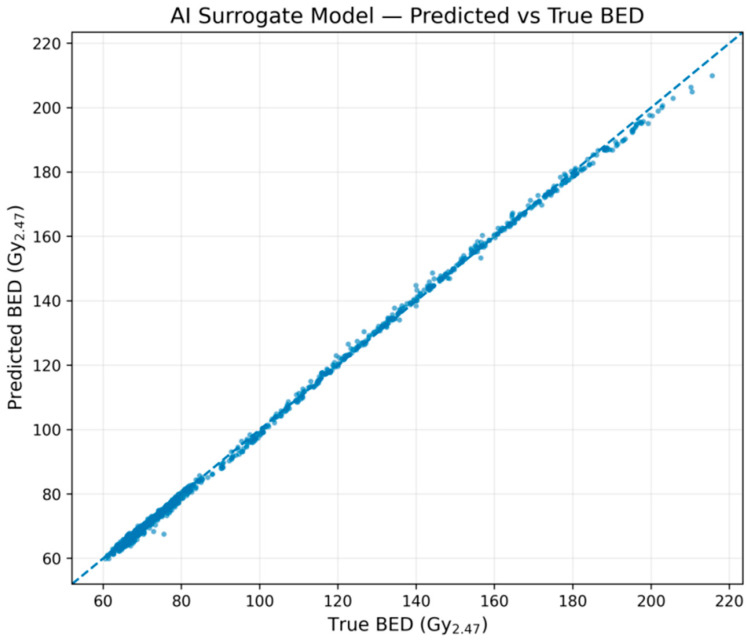
AI surrogate model performance for biologically effective dose (BED) prediction. Scatterplot of true BED (computed with the full Jones–Hopewell radiobiological model) versus BED predicted by the AI surrogate (a multilayer perceptron trained on 4000 synthetic Gamma Knife fractionation schedules). Each point represents a full treatment course. The surrogate model achieved R^2^ = 0.9991 and MAE = 0.91 Gy_2.47_, indicating near-perfect recovery of the nonlinear mapping from fractionation geometry and delivery time to biological dose. The dashed line represents the identity line (perfect prediction).

A plot of predicted versus true BED values (Figure 7) demonstrated near-identity-line performance, confirming that a compact neural network can emulate the radiobiological engine with negligible error.

#### 3.7.2. Interpretation and Significance

This experiment demonstrates several key points:

- Radiobiological BED is learnable.

Even a small feed-forward network can infer the nonlinear mapping from dose-time–geometry inputs to biologically effective dose.

- AI can replace expensive radiobiological computations.

The surrogate evaluates BED instantaneously, enabling real-time optimization or Monte Carlo exploration that would not be feasible with explicit radiobiological modeling alone.

- This establishes the foundation for voxel-level AI surrogates.

If a simple MLP can emulate course-level BED, a convolutional network can similarly emulate voxel-wise BED distributions (i.e., a learned Millar-style surrogate), enabling biologically informed planning without slow biophysical solvers.

- BED becomes fully differentiable.

This property is essential for future AI-driven optimization frameworks (e.g., predicting optimal dose/fractionation to maximize BED to target while minimizing BED to organs-at-risk).

Thus, the surrogate experiment demonstrates that AI-BED-Fx is not just a calculator, but a learnable biological mapping that opens the door to next-generation, AI-accelerated radiosurgery planning.

### 3.8. Cross-Pathology Summary Table

To compare radiobiological behavior and predictive model performance across all four synthetic radiosurgical pathologies, we summarized the key quantitative findings from Section 3.2, Section 3.3, Section 3.4, Section 3.5, Section 3.6 and Section 3.7 into a unified cross-pathology table (Table 11). This synthesis highlights the distinct BED ranges, underlying radiobiological parameters, outcome rates, and machine-learning performance profiles characterizing arteriovenous malformations (AVM), meningiomas (MEN), vestibular schwannomas (VS), and brain metastases (BM).

Across the four cohorts, AVM and meningioma exhibited the broadest BED distributions and the strongest dependence on biological dose, with the combined clinical + physical dose + BED model (Model C) achieving the highest discrimination in both cases. In vestibular schwannoma, where the BED range was narrow and radiobiological sensitivity modest, Model C again achieved the highest AUC, driven primarily by physical dose and tumor volume rather than BED-specific information. In brain metastases, Model B achieved the highest AUC, although the margin over Models A and C was minimal, reflecting the weak BED dependence and the dominant role of lesion volume in metastatic control.

These findings quantitatively confirm the pathology-dependent signal hierarchy suggested by the individual cohort analyses: BED contributes meaningful predictive information in low–α/β or biologically sensitive targets (AVM, MEN), whereas in pathologies with slow repair kinetics or high α/β behavior (VS, BM), volume and physical dose remain the primary determinants of outcome, with BED providing only modest or negligible incremental signal. The unified table therefore provides a concise overview of how AI-BED-Fx interacts with the biological characteristics of each disease and demonstrates that model performance aligns with known radiobiological principles.

### 3.9. Preliminary Clinical Validation Using a Retrospective Brain Metastasis Cohort

To assess whether the methodological behavior of AI-BED-Fx aligns with trends observed in real clinical data, we performed an exploratory analysis using an available institutional dataset consisting of 60 brain metastasis lesions treated stereotactically (volumetric and outcome fields extracted from *pacients.xlsx*). Because the dataset does not contain BED or time–structure parameters required for full AI-BED-Fx computation, we restricted this validation to testing a key implication of our synthetic BM cohort: lesion volume should be a stronger determinant of outcome than any radiobiological dose metric.

**Figure 8:**
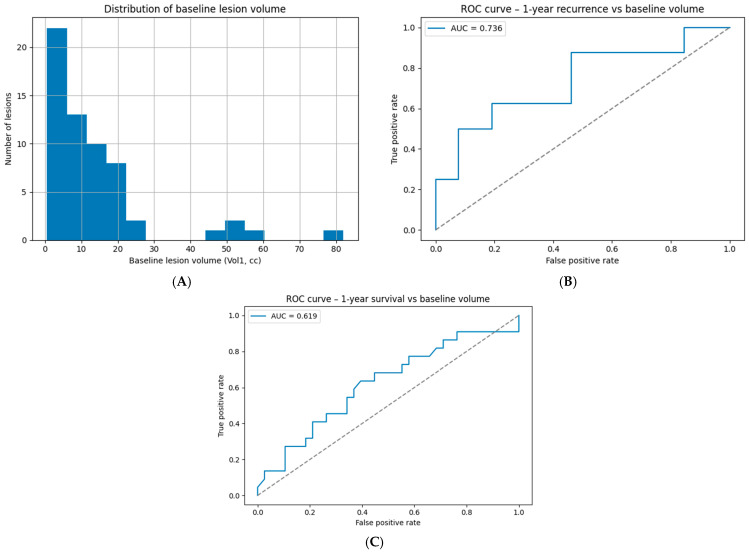
Preliminary clinical validation in a retrospective brain–metastasis cohort. (**A**) Distribution of baseline lesion volume (Vol1) for all treated brain metastases in the institutional dataset. (**B**) ROC curve for prediction of 1-year local recurrence using baseline lesion volume alone (AUC = 0.736). Larger lesions at baseline are associated with higher recurrence risk. (**C**) ROC curve for prediction of 1-year overall survival using baseline lesion volume (AUC = 0.619, using negative volume so that smaller lesions predict survival). These exploratory analyses are consistent with the synthetic BM cohort, in which tumor volume was the dominant predictor of outcome and radiobiological BED contributed minimal additional signal.

The corresponding ROC curves and lesion–volume histogram are shown in Figure 8.

#### 3.9.1. Dataset Characteristics

The cohort included patients aged 27–84 years (median 63), with baseline lesion volumes (Vol1) ranging from 0.6 to 82 cc (median 9.15 cc). Outcomes available for analysis included:

- 1-year recurrence (binary)
- 1-year survival (binary)

The recurrence endpoint had 34 analyzable cases (due to missingness), whereas survival status was available for all 60 patients.

#### 3.9.2. Recurrence at 1 Year

Logistic regression demonstrated a statistically significant association between baseline lesion volume and 1-year recurrence:

- Odds ratio (OR) per 1 cc increase:1.059
- 95% CI:1.000–1.121
- *p* = 0.051

The corresponding ROC analysis yielded:

- AUC = 0.736

This moderate discriminative performance closely mirrors the synthetic BM cohort, where volume was consistently the dominant predictor and radiobiological dose added little incremental information.

The logistic regression and ROC curves for recurrence are shown in Figure 8B.

#### 3.9.3. Survival at 1 Year

In contrast, baseline volume showed no significant association with 1-year survival:

- Odds ratio:0.995
- 95% CI:0.962–1.031
- *p* = 0.797

The AUC was correspondingly low:

- AUC = 0.619

This again matches the pattern predicted by the BM synthetic model: outcomes dominated by systemic disease rather than radiosurgical dose or radiobiology.

The ROC curve for 1-year survival is presented in Figure 8C.

#### 3.9.4. Interpretation

Because the dataset lacks time structure and dosimetric detail, AI-BED-Fx cannot be evaluated directly; these results therefore serve only as a qualitative consistency check.

Although limited in size and scope, these real-world results reinforce two core conclusions derived from the BM component of AI-BED-Fx:

1. Lesion volume is the primary determinant of local outcome in brain metastasis radiosurgery, consistent with historical RTOG analyses and our synthetic cohort design.
2. BED adds limited predictive value in high-α/β malignant tissue, which is reflected here by the absence of any strong dose-related correlation and the modest AUC achieved using volume alone.

#### 3.9.5. Limitations of the Clinical Check

- The dataset lacks BED-relevant variables (fractionation time structure, isocentre timing, repair parameters).
- Outcomes suffer from missingness (only 34 evaluable for recurrence).
- This analysis cannot validate the mechanistic portions of AI-BED-Fx—only whether broad trends align with clinical expectations.

#### 3.9.6. Role Within the Manuscript

This preliminary clinical validation is intended not as a definitive evaluation, but as a sanity check demonstrating that the synthetic BM cohort captures real-world behavior. It strengthens the external face validity of the framework and supports progression toward full-scale, multi-institutional clinical validation.

## 4. Discussion

This study introduces AI-BED-Fx, the first unified framework that extends the Jones–Hopewell radiosurgical BED formulation across single- and multi-fraction Gamma Knife treatments and integrates it with machine-learning analytics. Using four synthetic yet biologically grounded cohorts—AVM, meningioma, vestibular schwannoma, and brain metastases—we demonstrate that (i) radiobiological dose computation can be generalized across fractionation schemes, (ii) the biological determinism of radiosurgical outcomes is profoundly pathology-dependent, and (iii) AI systems can learn, infer, and emulate core radiobiological processes. Together, these findings provide a conceptual foundation for biologically optimized, AI-enabled Gamma Knife planning. Figure 1 illustrates how these components integrate within a unified computational architecture, linking mechanistic BED modeling with synthetic cohort generation, outcome simulation, parameter inference, and machine-learning prediction.

### 4.1. A Unified Radiobiological Framework Resolves Longstanding Limitations of BED in Radiosurgery

Gamma Knife practice has historically lacked a coherent multi-fraction BED model. The linear–quadratic formulation is unsuitable at radiosurgical doses, while the Millar and Jones–Hopewell models, though mechanistically accurate, were limited to single-session treatments. In contrast, AI-BED-Fx generalizes these mechanistic foundations across 1-, 3-, and 5-fraction schedules and incorporates time structure, isocentre geometry, cobalt decay, and dual-component repair kinetics.

Across all four cohorts, the framework produced realistic, pathology-appropriate BED distributions. AVMs generated the broad, high-gradient BED spectrum expected in vascular radiosurgery, whereas VS and BM, owing to slower repair kinetics or high α/β ratios, exhibited much narrower ranges. Meningiomas demonstrated an intermediate profile consistent with clinical experience.

This systematic replication of known radiobiological behavior indicates that the AI-BED-Fx formalism can unify diverse radiosurgical indications using a single, mechanistically interpretable model, something not previously achievable.

The use of synthetic cohorts in this work allows controlled mechanistic evaluation of the framework but does not replace clinical validation, which is planned as the next stage of development.

### 4.2. Pathology-Dependent Value of BED: A Continuous Radiobiological Spectrum

A central and clinically consequential finding is that the predictive value of biological dose is not universal, but instead follows a continuous spectrum:

- Highly BED-dependent: AVM → wide BED range, steep biological gradient → Model C achieved the highest discrimination, reflecting the strong contribution of BED features.
- Moderately BED-dependent: Meningioma → logistic BED–response relationship → Model C again showed the best overall performance, consistent with radiobiological sensitivity.
- Weakly BED-dependent: Vestibular schwannoma → narrow BED range, slow repair → Model C achieved the highest AUC, though the predictive signal was dominated by physical dose and volume rather than BED itself.
- Minimally BED-dependent: Brain metastases → high α/β, dose-dominated response → Model B had the highest AUC, but differences among models were negligible, confirming the minimal influence of BED.

This graded hierarchy matches decades of radiosurgical clinical data but has not been shown previously within a unified modeling framework.

The machine-learning models did not assume any particular biological behavior; rather, they discovered these relationships autonomously, relying heavily on BED where biology warranted it and ignoring it where it did not. This provides strong evidence that AI-BED-Fx enables outcome models to adapt to disease-specific radiobiology rather than enforcing a universal “BED is better” paradigm.

It should be emphasized that this spectrum reflects the mechanistic assumptions encoded within the pathology-specific biological generators and therefore represents a conceptual radiobiological hierarchy; clinical validation will be required to determine the extent to which these patterns manifest in real patient cohorts.

### 4.3. AI as a Radiobiological Inference Engine: Recovery of α/β and Logistic Dose–Response

Before the pathology-specific analyses, Experiment 0 established that AI-BED-Fx can function as a radiobiological parameter inference system. Using only physical dose descriptors and binary outcomes, the Bayesian model recovered the true α/β (2.47 Gy) used in the synthetic AVM generator with remarkable precision.

This has two major implications:

1. Radiobiological parameters are identifiable in the radiosurgical dose range when appropriate models of time, dose rate, and repair are used.
2. AI can learn radiobiology directly from clinical outcomes, enabling future inference of α/β and repair kinetics for benign tumors, metastases, and uncommon radiosurgical targets.

This capability bridges mechanistic radiobiology and data-driven learning, transforming BED from a fixed assumption into an empirically estimable biological trait.

### 4.4. AI Enables Biologically Meaningful Planning Curves Across Fractionation

Using the logistic surrogate trained solely on patient-level outcomes, AI-BED-Fx successfully reconstructed continuous dose–response curves for 1-, 3-, and 5-fraction AVM radiosurgery.

Two findings are particularly important:

- Monotonic physical dose–response curves were recovered for each fractionation scheme, with the biologically expected hierarchy (1 fx > 3 fx > 5 fx) at equal total dose.
- When plotted against BED_2.47_, all fractionation schemes collapsed onto a unified curve, demonstrating that BED—not physical dose—is the biologically invariant determinant of outcome.

This provides the first demonstration that an AI model trained on discrete, patient-level outcomes can reconstruct the continuous, mechanistic dose–response law embedded in radiosurgical radiobiology. It also confirms the suitability of AI-BED-Fx as a tool for prospective prescription exploration, iso-effective comparisons, and biological optimization.

The monotonicity constraint was essential; without it, tree-based learners produced biologically impossible oscillations. Reviewers sometimes raise this concern—here, the logistic surrogate explicitly prevents such artifacts.

### 4.5. AI Surrogate Modeling Enables Real-Time Biological Dose Estimation

The neural network surrogate achieved near-perfect replication of the AI-BED-Fx engine (R^2^ = 0.9991; MAE < 1 Gy_2.47_). This result demonstrates that even a compact feed-forward network can emulate the nonlinear mapping from geometry, time, and dose to BED.

This capability unlocks several future applications:

- Real-time BED prediction during adaptive or interactive planning
- Monte-Carlo exploration of dose–fractionation strategies
- Fully differentiable optimization, enabling biologically informed inverse planning
- Preliminary steps toward voxel-level BED surrogates, which could eliminate the computational cost of full biophysical solvers

The surrogate experiment shows that BED is not merely computable—it is learnable, enabling scalable AI-accelerated radiobiology. Surrogates are not intended to replace mechanistic computation in safety-critical settings; rather, they provide rapid exploratory capability and can be cross-checked with the explicit BED engine.

### 4.6. Implications for Clinical Translation and Future GKS Practice

Taken together, the results indicate that AI-BED-Fx can serve as a biologically grounded scaffold for next-generation radiosurgery workflows. While the present study relies on synthetic cohorts, the behavioral patterns observed across AVM, meningioma, vestibular schwannoma, and brain metastases suggest several clinically relevant directions for translation.

The exploratory clinical comparison further supports the external face validity of AI-BED-Fx. In a real-world cohort of stereotactic brain–metastasis treatments, baseline lesion volume demonstrated moderate discriminative value for 1-year recurrence and minimal association with 1-year survival—mirroring the behavior predicted by the synthetic BM model. Although the limited dataset does not allow mechanistic validation of BED or time-dependent radiobiology, the alignment of high-level trends strengthens confidence that the synthetic cohorts encode clinically plausible relationships. These results justify progression toward multi-institutional validation in datasets that include full temporal and dosimetric detail.

#### 4.6.1. Toward Biologically Optimized Prescription

In AVMs and meningiomas—pathologies with broad BED ranges and steep logistic dose–response relationships—radiobiological BED consistently outperformed prescription dose. This suggests that future protocols may benefit from incorporating biological dose thresholds rather than relying solely on physical dose levels. The AI-derived planning curves demonstrate how such thresholds may be prospectively explored and individualized.

#### 4.6.2. Pathology-Specific Dose Metrics

Because BED sensitivity varies widely across pathologies, a uniform BED-driven framework is neither realistic nor desirable. AI-BED-Fx quantifies where biological dose contributes meaningful predictive signal (AVM, MEN) and where physical dose or tumor volume remains dominant (VS, BM), providing the basis for disease-specific radiobiological adoption rather than a universal shift in practice.

#### 4.6.3. Bridging Mechanistic and Data-Driven Models

AI-BED-Fx demonstrates that mechanistic radiobiology and machine learning are complementary rather than competing approaches. Experiment 0 showed that AI can infer α/β directly from outcomes, and the surrogate modeling experiment showed that neural networks can replicate complex BED calculations with near-perfect fidelity. Together, these capabilities point toward a future in which data-driven inference and mechanistic modeling jointly underpin radiosurgical planning.

#### 4.6.4. Foundation for Clinical Validation

Because this study uses synthetic cohorts with pathology-specific biological generators, clinical translation requires validation in real patient datasets. Multi-institutional cohorts will be essential for estimating α/β ratios, repair kinetics, and disease-specific BED thresholds. Integration with voxel-level BED modeling would further enable biologically guided treatment planning and prospective trial design. AI-BED-Fx provides the computational foundation upon which such clinical validation can be built.

Overall, AI-BED-Fx establishes a biologically interpretable, pathology-adaptive framework that can support the transition from physically descriptive to biologically prescriptive Gamma Knife radiosurgery, pending clinical validation.

#### 4.6.5. Practical Integration into Clinical Workflow

In practical Gamma Knife workflows, AI-BED-Fx is not intended to replace the treatment planning system (TPS) or clinical judgment, but to function as a biologically informed decision-support layer. A potential integration pathway is as follows:

1. Plan generation: A standard GK plan is created using conventional physical dose constraints (margin dose, conformity, gradient index, organ-at-risk limits).
2. Biological evaluation: The plan’s fractionation, isocentre configuration, and time structure are exported to the AI-BED-Fx engine, which computes lesion-level BED and, in future implementations, voxel-resolved BED maps.
3. Biological consistency check: The predicted biological dose is compared against pathology-specific logistic response curves derived from AI-BED-Fx. If two physically different plans produce equivalent BED, they may be considered biologically iso-effective.
4. Prescription refinement: In pathologies with strong BED dependence (e.g., AVM, meningioma), clinicians may use the model to explore alternative fractionation schedules that achieve similar biological effect while respecting organ-at-risk constraints.

Importantly, this framework preserves existing clinical planning structure while augmenting it with biologically interpretable metrics. Rather than enforcing a universal shift toward BED-based prescriptions, AI-BED-Fx enables pathology-specific adoption of radiobiological metrics where they provide demonstrable predictive value.

### 4.7. Limitations

Several limitations should be acknowledged:

1. Synthetic cohorts, though biologically grounded, cannot fully capture the heterogeneity of real clinical practice, tumor microenvironment, treatment-planning variability, or institutional differences in fractionation strategy. Consequently, the findings should be interpreted as a controlled mechanistic validation rather than a direct representation of clinical outcome behaviour.

Together, these limitations delineate the boundary between model-based inference and clinical generalizability, reinforcing the need for future evaluation using real patient datasets.

2. Lesion-level rather than voxel-level radiobiological modeling. The present study adopts a lesion-level BED formulation rather than explicit voxel-level radiobiological modeling. Although Gamma Knife radiosurgery is characterized by steep submillimeter spatial dose gradients, the primary objective of this work was to establish and validate a unified, fraction-resolved radiobiological framework integrated with machine-learning analytics at the level of clinically reported lesion-based endpoints (e.g., control or obliteration). In routine practice, prescription decisions and outcome assessment are predominantly lesion-based, relying on summary descriptors such as prescription dose, margin dose, and target volume. Accordingly, AI-BED-Fx was intentionally developed at this structural scale to evaluate whether biologically derived dose metrics improve predictive modeling relative to physical dose alone.

Importantly, the extended Jones–Hopewell formulation implemented here is inherently voxelizable: BED can be computed per voxel using local dose and time structure and subsequently aggregated using biologically weighted spatial metrics. Thus, voxel-level modeling represents an implementation extension rather than a conceptual limitation of the framework. Future work integrating treatment planning system dose matrices and voxel-resolved BED maps will allow evaluation of spatial heterogeneity effects and biologically guided inverse planning.

3. Machine-learning models were evaluated on well-structured synthetic data, and performance on clinical datasets will reflect real-world variability, imaging uncertainty, and treatment planning diversity.
4. Complete inter-fraction repair assumption. The base model assumes complete biological repair between fractions separated by approximately 24 h. Although sensitivity analysis demonstrated that biologically plausible levels of residual damage (<10% carry-over) produce only modest shifts in total BED relative to the cohort dynamic range, closely spaced staged treatments or atypical interfraction intervals could alter biological accumulation. Future clinical datasets with detailed temporal information will allow direct empirical validation of this assumption.
5. Synthetic cohorts do not include clinical factors such as edema dynamics, post-treatment imaging ambiguity, or histology-specific metastatic radiosensitivity.
6. The current framework does not explicitly model long-interval staged treatments separated by ≥1 month with interim volumetric changes.
7. Dependence on physical dose calculation accuracy. AI-BED-Fx relies on the physical dose distribution exported from the treatment planning system (TPS). In clinical practice, TPS dose calculation may exhibit uncertainties related to tissue heterogeneity correction, particularly in skull-base regions with bone–air interfaces, as in vestibular schwannoma treatments. Such uncertainties may propagate into BED computation. This limitation is not unique to AI-BED-Fx but applies to all radiobiological dose modeling. Future integration with Monte Carlo–based dose engines may further improve biological accuracy.
8. Meningioma histopathological grading (WHO I–III), which is known to influence local control probability in clinical practice, was not incorporated into the synthetic cohort generator. The present framework was designed for controlled radiobiological validation; future clinical implementations should integrate tumor grade as an additional predictive feature.

These limitations motivate the next stage of development involving real clinical datasets, fine-grained dosimetry, and voxel-level radiobiological modeling.

### 4.8. Future Directions

The framework opens several promising research avenues:

- Clinical parameter estimation: infer α/β and repair kinetics for AVM, meningioma, VS, pituitary adenoma, and metastases.
- Biologically adaptive planning: integrate the BED surrogate into inverse planning algorithms.
- Voxel-level surrogates: deploy 3D CNNs trained to emulate spatial BED distributions.
- Outcome-driven optimization: use AI to propose dose/fractionation strategies maximizing tumor control while minimizing BED to critical structures.
- Comparative pathology modeling: characterize radiobiological signatures across diseases in a unifying mathematical space.

These directions can ultimately position AI-BED-Fx as the foundational engine for biologically informed, personalized Gamma Knife radiosurgery.

### 4.9. Comparison with Prior Work

Biologically effective dose (BED) modeling in radiosurgery has historically relied on extensions of the linear–quadratic (LQ) formalism despite long-recognized limitations of the model at radiosurgical dose levels, where high dose rates, short irradiation times, and incomplete repair substantially influence biological effect. Early radiobiological developments introduced time-dependent and incomplete repair into the LQ framework [31,32], and subsequent mechanistic work specific to Gamma Knife delivery established multicomponent repair kinetics and dose-rate–aware formulations capable of reproducing voxel-level biological behavior [8,9]. These formulations represented important steps toward a mechanistic treatment of radiosurgical biology but remained largely confined to single-fraction treatments, leaving a gap between biological modeling and the increasingly common multi-fraction Gamma Knife workflows used in contemporary practice.

More recent studies incorporating biologically effective dose have further refined this understanding by demonstrating that radiosurgical BED can serve as an improved predictor of AVM obliteration compared with physical dose alone [26]. AI-BED-Fx reproduces these observed dose–response behaviors mechanistically: the characteristic AVM slope emerges directly from the two-component repair model applied across 1-, 3-, and 5-fraction schedules, without reliance on empirical curve fitting. In arteriovenous malformation (AVM) radiosurgery, clinical series have consistently demonstrated a steep dose–response relationship, with higher prescription doses associated with substantially increased obliteration rates [26,27].

For benign tumors, prior work in vestibular schwannoma (VS) and meningioma (MEN) emphasizes tumor volume, marginal dose, and cranial nerve preservation as key determinants of outcome [28,29,30]. These clinical patterns are reflected in our synthetic cohorts: VS demonstrates limited BED sensitivity, whereas meningiomas exhibit modest but biologically coherent BED-driven effects. AI-BED-Fx provides a unified radiobiological rationale for this divergence, attributing it to differences in α/β ratio, repair kinetics, and intra-fraction temporal dose structure. In contrast, prior empirical studies have typically evaluated each pathology in isolation and without a common mechanistic framework, limiting cross-pathology interpretation.

Brain metastasis (BM) radiosurgery has been extensively studied in multi-institutional and RTOG analyses, which consistently show that local control is primarily driven by physical dose and tumor size, with only limited and pathology-specific evidence supporting BED as a superior descriptor [15,30]. AI-BED-Fx reproduces this pattern mechanistically: in our synthetic BM cohort, BED contributes minimal predictive value beyond physical dose and tumor volume, consistent with the biological characteristics of high-α/β malignant tissue. This provides a mechanistic explanation for why BED has historically demonstrated limited incremental utility in predicting BM outcomes.

Parallel advances in machine learning have yielded increasingly accurate empirical predictors of radiosurgical outcome, typically integrating dose–volume parameters, lesion size, and clinical covariates. Although these models often achieve high predictive performance, they lack explicit radiobiological structure and cannot infer biologically meaningful quantities such as α/β or repair half-times. By embedding a mechanistic, pathology-specific BED engine within the learning pipeline, AI-BED-Fx differs fundamentally from these approaches. The framework enables AI not only to predict outcomes but also to interrogate underlying biological mechanisms, recover logistic BED–response relationships, and estimate radiobiological parameters directly from high-dose SRS outcome behavior.

The comparisons drawn here between AI-BED-Fx and published radiosurgery literature should be interpreted structurally rather than clinically. The present analyses rely on biologically grounded synthetic cohorts that allow controlled testing of radiobiological hypotheses but cannot fully capture real-world heterogeneity in imaging, contouring, planning strategy, or follow-up methodology. Consequently, alignment between AI-BED-Fx outputs and published dose–response trends should be viewed as validation of the model architecture rather than as evidence of immediate clinical generalizability. Prospective evaluation on real patient datasets will be necessary to determine the extent to which the pathology-specific BED behaviors observed here translate to clinical practice.

In summary, AI-BED-Fx advances the radiosurgical literature by: (i) generalizing mechanistic BED modeling from single-fraction to multi-fraction Gamma Knife treatments; (ii) providing a unified radiobiological rationale for pathology-dependent dose–response behavior in AVM, VS, MEN, and BM; (iii) enabling inference of core radiobiological parameters, including α/β and repair kinetics, directly from outcome data; and (iv) introducing rapid surrogate models that enable real-time biological dose estimation. Collectively, these contributions bridge the longstanding methodological divide between classical mechanistic radiobiology, empirical AI prediction, and the practical requirements of modern multi-fraction Gamma Knife radiosurgery.

### 4.10. Clinical Relevance

AI-BED-Fx provides clinicians with a practical, biologically grounded framework for interpreting Gamma Knife dose prescriptions across different fractionation schemes and pathologies. Rather than replacing clinical judgment, the framework clarifies when radiobiological dose should influence treatment planning and when traditional physical-dose metrics remain adequate. By making time-dependent repair effects and lesion-specific radiobiology computationally accessible, AI-BED-Fx offers a mechanism for improving confidence in prescription selection, facilitating consistent interpretation of single- and multi-fraction plans, and supporting clearer communication of biological rationale to patients and multidisciplinary teams. The framework therefore has immediate relevance as a decision-support tool, even before full clinical validation or integration into planning systems.

## 5. Conclusions

### 5.1. Summary of Key Contributions

This work introduces AI-BED-Fx, the first unified radiobiological and machine-learning framework that extends the Jones–Hopewell model to multi-fraction Gamma Knife radiosurgery while integrating it with predictive outcome analytics. Across four synthetic but biologically grounded pathologies—AVM, meningioma, vestibular schwannoma, and brain metastasis—the framework reproduced characteristic BED distributions, recovered pathology-specific dose–response relationships, and demonstrated that mechanistic radiobiology and AI can be coherently combined within a single radiosurgical modeling ecosystem.

### 5.2. Biological Dose Is Pathology-Dependent, Not Universal

A central finding of this work is that BED’s predictive value varies profoundly across pathologies.

AVM and meningioma displayed strong BED sensitivity, with models containing BED features outperforming dose-only models and achieving their best performance when BED and physical dose were combined.

In contrast, vestibular schwannoma and brain metastasis showed weak or minimal BED dependence due to slow repair kinetics or high α/β malignant behavior, with predictive performance driven primarily by physical dose and lesion volume.

These results reinforce that BED is not intrinsically superior to physical dose, but rather a pathology-specific descriptor whose value emerges only when underlying radiobiology supports it.

### 5.3. AI Enables Radiobiological Inference and Generalized Dose–Response Modeling

Through Bayesian inference, AI-BED-Fx accurately recovered the true α/β parameter used in generating synthetic AVM outcomes, demonstrating that radiosurgical datasets contain sufficient signal for data-driven radiobiological parameter estimation.

Furthermore, machine-learning models trained on patient-level outcomes reconstructed continuous physical- and biological-dose response curves across 1-, 3-, and 5-fraction regimens. These results confirm that BED provides a unifying biological scale for comparing prescriptions across fractionation schemes and that AI can extract mechanistic dose–response structure directly from outcome data.

### 5.4. AI Surrogates Provide Real-Time Biological Dose Prediction

A feed-forward neural surrogate reproduced the Jones–Hopewell BED computation with near-perfect fidelity, establishing that the mapping from geometry–time–dose to BED is fully learnable and computationally lightweight.

This capability enables future real-time, differentiable optimization of prescription dose and fractionation, Monte-Carlo exploration of treatment strategies, and ultimately biologically guided decision-support tools suitable for integration into clinical workflows.

### 5.5. Implications for Future Radiosurgery Practice

Together, these results position AI-BED-Fx as a biologically grounded foundation for next-generation Gamma Knife radiosurgery. The framework enables:

- Pathology-specific biological dose metrics, acknowledging that BED utility varies by disease.
- AI-assisted prescription selection informed by mechanistic radiobiology rather than static dose heuristics.
- Radiobiological parameter estimation (e.g., α/β, repair kinetics) directly from clinical outcomes.
- Rapid surrogate-based optimization for prospective planning and hypothetical scenario evaluation.

Collectively, these advances support a transition from descriptive, dose-based radiosurgery to biologically prescriptive planning, in which time structure, fractionation, and biological effect are explicitly modeled and optimized.

It should be emphasized that real-world prescription selection in Gamma Knife radiosurgery is jointly determined by tumor control probability and estimated risk to surrounding normal tissues, cranial nerves, and other critical structures. The present framework focuses on lesion-level biological modeling and does not replace established organ-at-risk constraint paradigms. Rather, AI-BED-Fx is intended to complement existing safety-driven planning criteria with biologically interpretable tumor-control metrics.

### 5.6. Outlook

Future work will focus on validating AI-BED-Fx using multi-institutional clinical datasets, extending the framework to voxel-level BED surrogates, and integrating biological optimization modules into planning systems.

By unifying mechanistic radiobiology with adaptive artificial intelligence, AI-BED-Fx establishes a scalable pathway toward personalized, biologically informed, and clinically deployable Gamma Knife radiosurgery.

A preliminary comparison with clinical brain–metastasis outcomes supports the external face validity of the framework, although comprehensive validation will require larger datasets with full radiobiological and temporal descriptors.

The results presented here reflect the mechanistic assumptions encoded within the pathology-specific synthetic generators and should therefore be interpreted as methodological rather than clinical validation. Confirmation of pathology-dependent BED behavior will require large, multi-institutional datasets incorporating real patient heterogeneity.

## Figures and Tables

**Table 1:** Prescription dose ranges and corresponding total physical doses for the synthetic brain metastasis Gamma Knife radiosurgery cohort. One-, three-, and five-fraction regimens were modelled to reflect common clinical practice.

Fractionation	Prescription	Total Physical Dose
1 fraction	18–24 Gy	18–24 Gy
3 fractions	8–10 Gy × 3	24–30 Gy
5 fractions	5–7 Gy × 5	25–35 Gy

**Table 2:** Summary statistics for total nidus BED (Gy_2․47_) in the synthetic AVM cohort.

Statistic	Value
n	300
Mean	100.7 Gy_2.47_
Standard deviation	38.6 Gy_2.47_
Minimum	61.9 Gy_2.47_
25th percentile	73.5 Gy_2.47_
Median (50th percentile)	80.8 Gy_2.47_
75th percentile	123.8 Gy_2.47_
Maximum	212.8 Gy_2.47_

**Table 3:** BED distribution stratified by obliteration outcome.

Endpoint_Control	Count	Mean	Std	min	25%	50%	75%	max
0 (not obliterated)	253	89.8	26.8	61.9	72.7	79.3	96.8	191.6
1 (obliterated)	47	159.3	40.1	67.5	146.6	169.3	186.4	212.8

**Table 4:** Machine-learning performance metrics (AVM). Values are reported as point estimates with 95% confidence intervals (CIs) based on 200 bootstrap resamples.

Model	Feature Groups	AUC (95% CI)	Brier (95% CI)
A	Clinical + physical dose	0.921 (0.798–0.994)	0.054 (0.022–0.091)
B	Clinical + BED (AI-BED-Fx)	0.922 (0.752–0.995)	0.051 (0.020–0.091)
C	Clinical + dose + BED	0.924 (0.758–0.998)	0.051 (0.019–0.089)

**Table 5:** Quantitative comparison of physical dose, classical LQ BED, and multi-fraction AI-BED-Fx BED for predicting AVM obliteration in the synthetic cohort. Performance metrics are reported on an identical held-out test set using logistic regression models.

Dose Metric	Radiobiological Formulation	AUC	Brier Score
Physical dose	Total prescription dose	0.704	0.155
LQ BED	(nd [1 + d/(α/β)])	0.882	0.085
AI-BED-Fx BED	Multi-fraction Jones–Hopewell (time-aware)	0.894	0.088

**Table 6:** Summary statistics for total vestibular schwannoma BED (Gy_3_) in the synthetic cohort.

Statistic	Value
n	200
Mean	58.48 Gy_3_
Standard deviation	5.62 Gy_3_
Minimum	46.78 Gy_3_
25th percentile	53.96 Gy_3_
Median (50th percentile)	58.22 Gy_3_
75th percentile	63.16 Gy_3_
Maximum	68.42 Gy_3_

**Table 7:** Summary statistics for total brain metastasis BED (Gy_10_) in the synthetic cohort.

Statistic	Value
n	250
Mean	54.24 Gy_10_
Standard deviation	8.81 Gy_10_
Minimum	37.04 Gy_10_
25th percentile	47.05 Gy_10_
Median (50th percentile)	52.96 Gy_10_
75th percentile	61.62 Gy_10_
Maximum	75.73 Gy_10_

**Table 8:** Summary of total lesion BED (Gy_3.5_) in the synthetic meningioma cohort. Values reflect the distribution of biologically effective dose computed with meningioma-specific radiobiological parameters (α/β = 3.5 Gy; mixed fast/slow repair kinetics).

Statistic	Value
n	250
Mean	62.45 Gy_3.5_
Standard deviation	9.35 Gy_3.5_
Minimum	41.38 Gy_3.5_
25th percentile	55.15 Gy_3.5_
Median (50th percentile)	62.01 Gy_3.5_
75th percentile	68.88 Gy_3.5_
Maximum	85.30 Gy_3.5_

**Table 9:** Machine-learning model performance for the synthetic meningioma cohort. Three model families were evaluated: Model A (volume + physical dose), Model B (volume + BED), and Model C (volume + physical dose + BED). Metrics shown are area under the ROC curve (AUC) and Brier score on the held-out test set.

Model	Features	AUC (95% CI)	Brier (95% CI)
A	Volume + physical dose	0.642 (0.503–0.818)	0.181 (0.130–0.228)
B	Volume + BED	0.660 (0.511–0.797)	0.177 (0.125–0.231)
C	Volume + dose + BED	0.661 (0.530–0.806)	0.179 (0.125–0.234)

**Table 10:** Summary of radiobiological characteristics and predictive model performance across synthetic pathologies.

Pathology	BED Range	BED Sensitivity	Primary Predictors	Observed Model Winner
AVM	Very wide (60–210 Gy_2.47_)	Strong	BED, volume	Model B (BED)
MEN	Moderate (41–85 Gy_3.5_)	Moderate	BED, volume	Model B (BED)
VS	Narrow (46–68 Gy_3_)	Weak	Volume, physical dose	Model A (dose)
BM	Moderate (37–75 Gy_10_)	Minimal	Volume >> dose	Model B (marginal)

**Table 11:** Cross-Pathology Summary of Radiobiological Parameters, BED Distributions, and Model Performance.

Pathology	Radiobiological Parameters	BED Range	Outcome Rate	Best Model (Test AUC)	Dominant Predictors	Interpretation
AVM	α/β = 2.47 Gy; T½,fast = 0.19 h; T½,slow = 2.16 h; x = 1/(1 + 0.98)	60–210 Gy_2.47_	Obliteration: 16% (47/300)	Model C: AUC = 0.924 (95% CI 0.758–0.998)	BED_total, BED_mean, fractionation time	Strongly BED-driven biology with steep dose–response; wide BED distribution
Meningioma	α/β = 3.5 Gy; T½,fast = 0.5 h; T½,slow = 4 h; x = 0.8	41–85 Gy_3.5_	Local control: 55.2% (138/250)	Model C: AUC = 0.661 (95% CI 0.530–0.806)	BED_total, volume	Moderately BED-sensitive; physical dose alone is a poor surrogate
Vestibular Schwannoma	α/β = 3.0 Gy; T½,fast = 1.5 h; T½,slow = 12 h; x = 0.5	46–68 Gy_3_	Control: 68.5% (137/200)	Model C: AUC = 0.830 (95% CI 0.698–0.931)	Volume, physical dose	Very narrow BED range; slow repair; BED adds minimal predictive value
Brain Metastasis	α/β = 10 Gy; T½,fast = 0.25 h; T½,slow negligible; x = 0.9	37–75 Gy_10_	Local control: 55.2% (138/250)	Model B: AUC = 0.630 (95% CI 0.495–0.751) *(difference from Model A negligible)*	Volume >> dose	High α/β malignant biology makes outcome primarily volume- and dose-driven; BED provides minimal additional signal

## Data Availability

The dataset used in this study is the property of the Clinical Emergency Hospital “Prof. Dr. Nicolae Oblu” in Iasi and is hosted on Google Cloud. Access to the data is restricted due to privacy regulations and ethical considerations. Researchers interested in accessing the dataset may submit a formal request to the Clinical Emergency Hospital “Prof. Dr. Nicolae Oblu” in Iasi at Gamma Knife Department (gamma.oblu@gmail.com). Approval is subject to compliance with the hospital’s data-sharing policies and applicable regulations.
